# Non-coding RNA expression analysis revealed the molecular mechanism of flag leaf heterosis in inter-subspecific hybrid rice

**DOI:** 10.3389/fpls.2022.990656

**Published:** 2022-09-26

**Authors:** Mengyao Wang, Jianbo Wang

**Affiliations:** State Key Laboratory of Hybrid Rice, College of Life Sciences, Wuhan University, Wuhan, China

**Keywords:** inter-subspecific hybrid rice, heterosis, miRNA, lncRNA, circRNA, ceRNA

## Abstract

Heterosis has been used widespread in agriculture, but its molecular mechanism is inadequately understood. Plants have a large number of non-coding RNAs (ncRNAs), among them, functional ncRNAs that have been studied widely containing long non-coding RNA (lncRNA) and circular RNA (circRNA) that play a role in varied biological processes, as well as microRNA (miRNA), which can not only regulate the post-transcriptional expression of target genes, but also target lncRNA and circRNA then participate the competing endogenous RNA (ceRNA) regulatory network. However, the influence of these three ncRNAs and their regulatory relationships on heterosis is unknown in rice. In this study, the expression profile of ncRNAs and the ncRNA regulatory network related to heterosis were comprehensively analyzed in inter-subspecific hybrid rice. A total of 867 miRNAs, 3,278 lncRNAs and 2,521 circRNAs were identified in the hybrid and its parents. Analysis of the global profiles of these three types of ncRNAs indicated that significant differences existed in the distribution and sequence characteristics of the corresponding genes. The numbers of miRNA and lncRNA in hybrid were higher than those in its parents. A total of 784 ncRNAs (169 miRNAs, 573 lncRNAs and 42 circRNAs) showed differentially expressed in the hybrid, and their target/host genes were vital in stress tolerance, growth and development in rice. These discoveries suggested that the expression plasticity of ncRNA has an important role of inter-subspecific hybrid rice heterosis. It is worth mentioning that miRNAs exhibited substantially more variations between hybrid and parents compared with observed variation for lncRNA and circRNA. Non-additive expression ncRNAs and allele-specific expression genes-related ncRNAs in hybrid were provided in this study, and multiple sets of ncRNA regulatory networks closely related to heterosis were obtained. Meanwhile, heterosis-related regulatory networks of ceRNA (lncRNA and circRNA) and miRNA were also demonstrated.

## Introduction

Heterosis is a ubiquitous phenomenon in sexually reproducing organisms, which has been widely used in crops, and has achieved great social and economic benefits. To date, three genetic hypotheses for heterosis were proposed, which included dominance, overdominance and epistasis (Larièpe et al., 2012; Shen et al., 2014). Due to the complex molecular mechanisms, the current explanations for heterosis are still controversial (Xiong et al., 2021).

The yield of hybrid rice was 20–30% higher than conventional rice (Qian et al., 2021). The research on heterosis of hybrid rice is crucial for improving rice yield and has been the focus of scientists for many years (Shao et al., 2019; Xiong et al., 2021). Two traditional subspecies, *indica* (*Oryza sativa* L. subsp. *indica* Kato) and *japonica* (*O. sativa* L. subsp. *japonica* Kato), have different physiological traits, genomic and epigenetic differentiation (Liu et al., 2007; Lu et al., 2022). These differences lead to obvious heterosis in inter-subspecific hybrid rice, which has potential production and research value (Zhang, 2022).

With the rapid rise of modern genomics techniques, noncoding RNA or nonprotein-coding RNAs (ncRNA or npcRNA), once called genomic “dark matter,” has been discovered (Morris and Mattick, 2014; Yu et al., 2019). By targeting to corresponding DNA, RNA and proteins, ncRNAs participate in a variety of biological processes and molecular mechanisms in plants (Ohtani, 2017; Chand Jha et al., 2021). In rice, many experimental reports have indicated the importance of ncRNAs in growth, development, environmental response (Yuan et al., 2018; Luan et al., 2019; Wang et al., 2020; Zhai et al., 2020; Qiao et al., 2021). Regrettably, the molecular functions of ncRNAs in plant heterosis formation remain poorly understood.

According to the length and function, ncRNAs in plants can be into lncRNAs, circRNAs, miRNAs, ribosomal RNAs (rRNAs), transfer RNAs (tRNAs) and others. MiRNA is a kind of single-stranded endogenous ncRNA abundant in animal and plant kingdom, about 20–24 nt in length, which regulates gene expression by pairing with cognate mRNA bases to silenced mRNA (Voinnet, 2009). LncRNA is an ncRNA with a length of more than 200 nt, located in the nucleus and cytoplasm, which can be categorized into long intergenic ncRNA (lincRNA), intron ncRNA (incRNA) and natural antisense transcript (NAT). It is involved in molecular functions such as chromatin level modification, alternative splicing of mRNA and the regulation of post-translation (Bardou et al., 2014; Laurent et al., 2015; Budak et al., 2020). CircRNA is a kind of closed-loop single-stranded ncRNA produced by reverse splicing, which is associated with a variety of biological processes in eukaryotes (Guo et al., 2014; Wang et al., 2019; Zhang et al., 2020). Although more and more circRNA profiles have been analyzed in plants, the role of circRNAs in plants remains limited compared to the numerous advances made in animals (Lu et al., 2015; Dou et al., 2017; Pan et al., 2018; Han et al., 2020).

In addition to the regulation of target mRNA by miRNAs mentioned above, some lncRNAs and circRNAs can competitively bind miRNAs then influence the expression of target mRNAs, which were called endogenous target mimics (eTMs) or competing endogenous RNAs (ceRNAs). Osa-miR2118 triggers the cleavage of lncRNA, *Photoperiod sensitive male sterility 1* (*PMS1T*), and this interaction of miRNA-lncRNA can result in male sterility of photoperiod sensitive rice (Fan et al., 2016). Two lncRNAs, MSTRG.66289.1 and MSTRG.52515.5, have been experimentally verified as ceRNAs of osa-miR156 and osa-miR396 in rice, respectively, which involved in the fertility conversion (Wang et al., 2021c). A total of 4,517 pairs of circRNA/lncRNA–miRNA–mRNA interaction networks were studied in MH63, and these networks might be involved in growth, development, metabolism and so on (Zhou et al., 2021). Despite these prior miRNA–lncRNA or miRNA–circRNA network investigations, we still have an incomplete understand of ceRNA in inter-subspecific hybrid rice.

In this study, we provided a deep exploration of ncRNA expression profiles and inheritance patterns among an inter-subspecific hybrid rice, covering expression analysis of three types of ncRNAs, identification of heterosis-related ncRNAs, and regulation analysis of heterosis-related miRNAs on two other ncRNAs. The additive expression of heterosis-related ncRNAs was analyzed, and ceRNA networks of these heterosis-associated ncRNAs were also demonstrated. This research provided a more detailed view of ncRNAs and their contribution to heterosis.

## Materials and methods

### Material planting and collection

The inter-subspecific hybrid rice combinations used in this study were proved to have flag leaf heterosis in the hybrid ZY19 (Wang and Wang, 2022). ZY19 and its parents (Z04A, *japonica* maternal line; ZHF1015, *indica* paternal line) were planted at test field of Wuhan University, Wuhan, China. Fertilization and pest control were consistent with conventional field management. At heading stage, flag leaves of hybrid and its parents (three biological replicates of each variety) were collected, frozen in liquid nitrogen for 5–6 h and stored at −80°C for RNA extraction.

### RNA extraction and quality detection

RNAs from the 9 sample of flag leaves were extracted by RN40 (Aidlab Biotechnologies, Beijing, China). NanoDrop 2000 spectrophotometer (Thermo Fisher Scientific, Wilmington, DE, United States) and Agilent Bioanalyzer 2,100 (Agilent Technologies, Santa Clara, CA, United States) detected RNA concentration, purity, and integrity. High-quality RNAs (concentration ≥ 313.9 ng/μl, RNA integrity number > 7.2) were used to prepare libraries needed for further sequencing.

### Preparation of rRNA-depleted strand-specific RNA (ssRNA) library and sequencing

The rRNA-depleted ssRNA libraries were used to identify mRNAs, lncRNAs, and circRNAs. The extracted high-quality RNAs were used as materials, and Ribo-off^®^ rRNA depletion kit (Vazyme, Nanjing, China) was used to remove rRNA. The first-strand cDNA was synthesized after fragmenting the rRNA-depleted RNA. Then, the second-strand cDNA was synthesized and purified. After purification, base A and adaptor were added to the 3′ end. By PCR amplification and quality detection, nine libraries were constructed for sequencing, which was performed on Illumina NovaSeq 6,000 Platform (Illumina, San Diego, CA, United States) to generate pair-ended 150 bp reads.

### Transcriptome assembly and lncRNA, circRNA identification

Prior to assembly, low-quality reads were removed from the raw reads of fastq format. After sequencing quality control, a total of 64.14 Gb (Q30 > 94.15%) of clean data were obtained. Clean reads were mapped onto Nipponbare (MSU Rice Genome Annotation Project Release 7, MSU-V7.0)^1^ by Hisat2. Finally, String Tie was used to compare reads on pairs for transcription splicing and quantification.

Transcripts annotated with Gffcompare were obtained, and unknown transcripts with length greater than 200 nt and exon number greater than two were used to screen for putative lncRNAs. From these transcripts, Coding Potential Calculator (CPC), Coding-Non-Coding Index (CNCI), Protein family (Pfam), and Coding Potential Assessment Tool (CPAT) were further used to obtain RNA with no coding ability. As well as the different types of lncRNAs including lincRNA, antisense lncRNA, sense lncRNA, and intronic lncRNA were distinguished. Based on the interaction mechanism between lncRNAs and their target genes, there were two methods to predict target genes of lncRNAs. On the one hand, since lncRNA can regulate the expression of their adjacent mRNA, the mRNAs within 100 kb of corresponding lncRNAs were designated as *cis-*target genes. On the other hand, lncRNA interacts with target mRNA through base complementation and predicted by LncTar.

The data obtained by ssRNA sequencing were used to predict circRNA by Findcirc software. Since the circRNA splicing site could not be directly mapped onto the reference genome, 20 bp at each end of unmapped reads was taken as anchor, which will be compared to the genome as an independent unit. After finding the binding position of circRNAs, if the sequences on both sides were GT/AG, they were judged to be circRNAs. Candidate circRNA screening pipeline was developed as follows. (1) GU/AG appeared on both sides of the splicing site. (2) Specific breakpoints. (3) Only two mismatches. (4) Maximum length of breakpoint was 2 nt. (5) The corresponding junction was supported by two reads at least. (6) Mapping onto the correct short sequence scored more than 35 points higher than mapping onto other positions. Finally, candidate circRNAs were identified.

### Small RNA library construction and sequencing

Using total RNAs extracted as input materials, nine sequencing libraries were generated using the VAHTS Small RNA Library Prep Kit for Illumina (Vazyme, Nanjing, China). The 3′ and 5′ ends of small RNA were connected to universal adapters, respectively, and then were amplified by reverse transcription and PCR. The target fragments were screened by gel separation techniques, and small RNA libraries suitable for Illumina platform were obtained. NovaSeq6000 (Illumina, San Diego, CA, United States) was used for small RNA sequencing after library quality inspection.

### miRNA identification and target prediction

The small RNA sequencing original data quality control process was as follows: (1) Removal of the joint. (2) Removal of sequences shorter than 18 nt or longer than 30 nt. (3) Removal of sequences with low-quality values for each sample. (4) Remove reads with N (N is an unidentifiable base) greater than 10%. Using Bowtie software, the clean reads obtained were compared with Silva database, GtRNAdb database, Rfam database and Repbase database for short sequences. SnoRNA, rRNA, tRNA and repeat sequences were filtered out to obtain unannotated reads containing miRNA. Then these obtained reads were mapped onto Nipponbare (MSU-V7.0) by Bowtie and obtained mapped reads. Then the sequences were compared with miRBase (V22) to identify the known miRNA. The novel miRNAs were predicted by miRDeep2. TargetFinder predicted miRNA targets based on known and novel miRNA and corresponding species genomic information.

### Differential expression and non-additive expression analysis

Differential expression analysis of four types of RNAs in three varieties (three biological replicates per variety) was analyzed by DESeq2. Fold Change (FC) represents the expression ratio between two varieties and *p* value of the original hypothesis represents the probability of expressing indifference. The identification of differentially expressed genes (DEGs), differentially expressed lncRNAs (DElncRNAs), differentially expressed circRNAs (DEcircRNAs) and differentially expressed miRNAs (DEmiRNAs) was based on the following thresholds: $|\log_{2}\mathrm{fold change}|>1$ and *p* < 0.05. The differentially expressed RNAs between hybrid and parents (including DEmRNA_HP_, DEmiRNA_HP_, DElncRNA_HP_, and DEcircRNA_HP_) were speculated to be involved in heterosis. Correspondingly, the differentially expressed RNAs between two parents were represented by DEmRNA_PP_, DEmiRNA_PP_, DElncRNA_PP_, and DEcircRNA_PP_.

Additive and non-additive ncRNA expression analysis was performed by comparing the ncRNA expression levels of hybrids with mid-parent value (MPV). The thresholds to identified non-additive ncRNAs were:


$$|\log_{2}\left(\begin{array}{l} \mathrm{ncRNA expression of}\;MPV\\ /\mathrm{ncRNA expression in}\;F1\;\mathrm{hybrid} \end{array}\right)|>1\;\mathrm{and}\;p\;<\;0.05.$$

### Heterosis-related ncRNA–mRNA and ncRNA–ncRNA integration analysis

To obtain heterosis-related ncRNA-mRNA regulatory network, we analyzed the regulatory relationship between ncRNAs and their corresponding targeted allele-specific expression genes (ASEGs) in hybrid. The ASEGs data are derived from previous studies (Wang and Wang, 2022) of the same inter-subspecific hybrid rice in this study. DEmRNA_HP_s corresponding to DEmiRNA_HP_s and DElncRNA_HP_s were analyzed. Meanwhile, DElncRNA_HP_s and DEcircRNA_HP_s regulated by DEmiRNA_HP_s were also analyzed. These networks were visualized using Cytoscape.

Using weighted correlation network analysis (WGCNA) in R software, the correlation coefficient between non-additive expressed ncRNAs and DEmRNA_HP_s was evaluated. Correlation coefficient with corresponding > 0.8 (positive) or < 0.8 (negative) was considered to be a co-expressed mRNA of non-additive expressed ncRNAs.

### ncRNA expression level validation

The results of the ssRNA and miRNA sequencing were validated by quantitative real-time PCR (qRT-PCR). A total of 10 lncRNAs, 5 circRNAs, and 5 miRNAs were randomly selected for verification. Total RNA was extracted from nine flag Leaf samples (including three biological replicates) using TRIzol reagent (Catalog No.15596–026, Invitrogen, Carlsbad, CA, United States). LncRNAs and circRNAs were verified using cDNA obtained by reverse transcription with random primers. According to the sequence of miRNA, cDNA synthesized with reverse transcription primer with stem-loop was used for quantitative verification of miRNA. SYBR (PowerUp^™^ SYBR^™^ Green Master Mix, Thermo Scientific^™^, United States) were used on an ABI Step One Plus Real-Time PCR System. *OsActin1* was a housekeeping gene for quantitative validation of lncRNA and circRNA. Forward primers and reverse universal primers for miRNA were designed according to the sequences of miRNA and reverse transcription primers, and U6 was used as housekeeping gene. The results were analyzed by comparative Ct (2^–ΔΔCt^).

## Results

### ncRNA and mRNA characteristics in the inter-subspecific hybrid rice combination

A total of 110 million clean reads were obtained from 9 small RNA libraries (Supplementary Table 1). Through mapping to the miRBase (V22) database and prediction by miRDeep2, 867 miRNAs were identified, containing 345 novel miRNAs and 522 known miRNAs (Table 1). Among these identified miRNAs, 514 miRNAs belonged to 153 gene families. To profile lncRNAs, circRNA and mRNA in each sample, ssRNA libraries were successfully constructed using the same plant material used for miRNA sequencing. After filtering low-quality reads, an average of 47.96 million reads were generated in each sample (Supplementary Table 1), and 88.98–91.90% of the clean reads were mapped uniquely to a single best location in the reference genome. Then a total of 3,278 lncRNAs were identified through basic screening and coding ability screening, including lincRNA 1789 (54.58%), antisense lncRNA 955 (29.13%), sense lncRNA 315 (9.61%), and intronic lncRNA 219 (6.68%; Table 1). Find_circ was used to predict the total circular RNA of three varieties, and 2,521 circRNAs were identified for subsequent analysis (Table 1). The mRNA expression levels were also profiled by lncRNA sequencing and the number of identified mRNAs was shown in Table 1. Furthermore, qRT-PCR was used to verify the relative expression levels of the three types of ncRNAs identified among different varieties, which indicated that the sequencing results were reliable (Supplementary Figure 1).

**Table 1:** Number of RNA identified in the inter-subspecific hybrid rice combination.

Different types of RNA		Z04A	ZHF1015	ZY19	Total
miRNA	known miRNA	471	427	472	522
	novel miRNA	343	338	345	345
lncRNA	intronic lncRNA	164	177	185	219
	antisense lncRNA	676	708	765	955
	sense lncRNA	238	231	243	315
	lincRNA	1,113	1,113	1,308	1789
circRNA		913	1,106	1,059	2,521
mRNA		29,903	29,091	30,450	36,260

**Figure 1:**
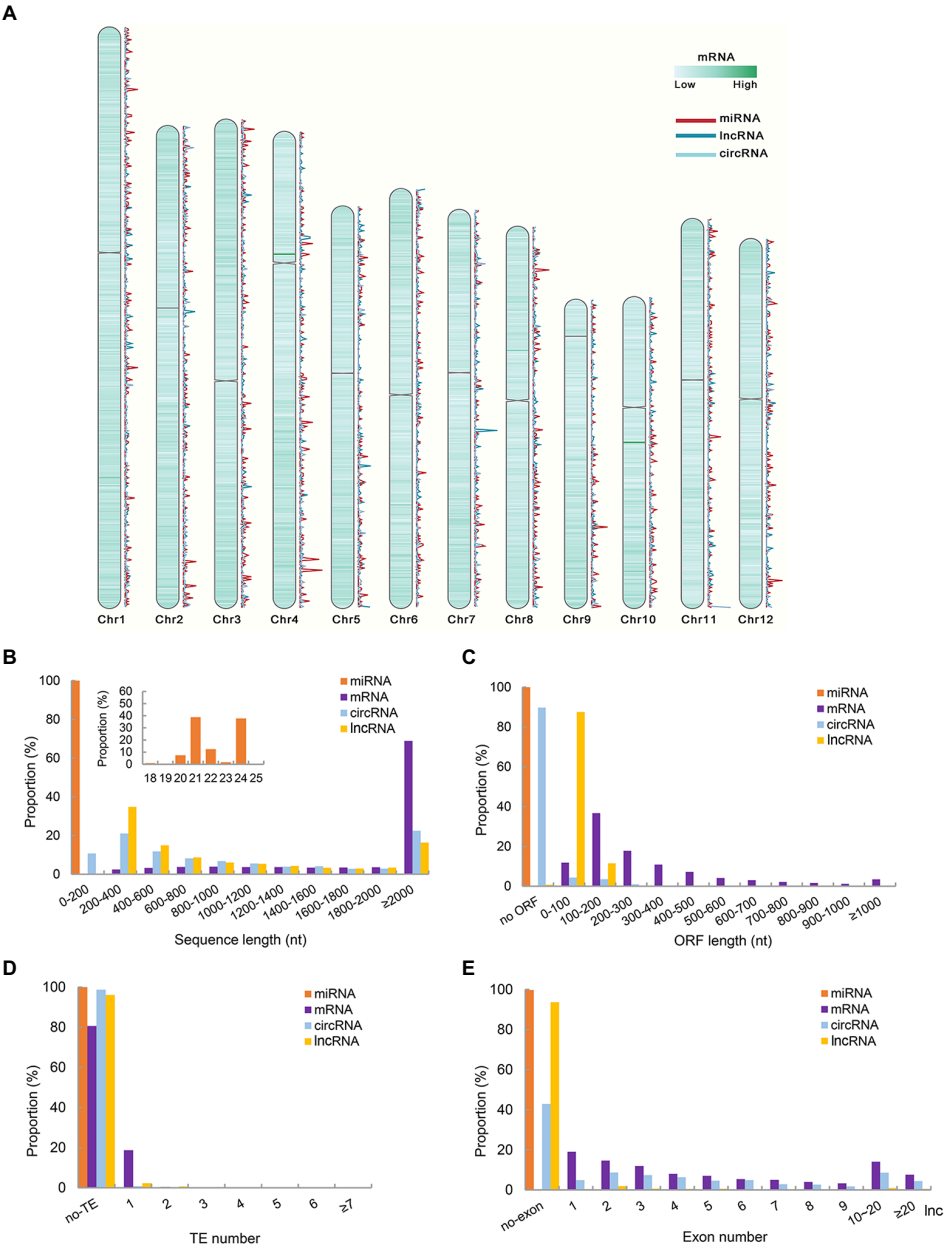
Genomic characteristics and structure characteristics of all identified RNAs corresponding genes. **(A)** Density map of mRNA, miRNA, lncRNA and circRNA corresponding genes on different chromosomes. **(B)** Sequence length of identified ncRNAs and mRNAs. **(C)** Corresponding DNA sequence ORF length of identified ncRNAs and mRNAs. **(D)** The number of TE contained in the corresponding DNA sequence of ncRNAs and mRNAs. **(E)** The number of Exon contained in the corresponding DNA sequence identified ncRNAs and mRNAs.

To investigate the differences in chromosome distribution, sequence and structure of different types of RNAs corresponding genes, the characteristics of the corresponding DNA sequences of the identified RNAs were compared in this study. Analysis of genomic characteristics of corresponding genes of RNAs showed that the proportion of all identified RNA corresponding sequences on chromosome 1 was higher than that on other chromosomes, followed by chromosome 2, and this distribution pattern might be related to chromosome length (Supplementary Figure 2A). Density statistics of all identified mRNA, miRNA, lncRNA, and circRNA corresponding genes were carried out with 10,000 bp sliding window. The results showed that the corresponding genes of the four types of RNAs were distributed in low density near the centromere on each chromosome (Figure 1A). The density distribution of miRNA and circRNA corresponding genes on each chromosome was similar, but there were many differences in the distribution of miRNA and lncRNA genes on each chromosome (Figure 1A). Furthermore, the proportion of genes corresponding to different types of RNA on each chromosome was calculated. mRNAs corresponding genes accounted for the highest proportion on each chromosome, followed by lncRNAs and circRNAs (Supplementary Figure 2B).

The transcript length of miRNA fragments was 18–25 nt, and the number of miRNAs with transcript lengths of 21 and 24 nt was the majority (Figure 1B). Compared with mRNAs, the transcript length of lncRNAs and circRNAs was mainly distributed in the range of 200–400 nt, which was much smaller than that of mRNA. This characteristic was consistent with previous studies on lncRNA in young panicles of rice (Wang et al., 2021c). At the same time, referring to the study on exons, ORF length and TE number of genes corresponding to lncRNAs in young panicles (Wang et al., 2021c), these elements of genes corresponding to 4 types of RNAs were analyzed and counted. MiRNAs corresponding DNA sequences were too short to identify ORF. The ORF length of most lncRNAs and circRNA corresponding genes was less than 200 nt, and 87.5% ORF of the lncRNAs corresponding genes was less than 100 nt, while the ORF length of most mRNAs corresponding genes was greater than 100 nt (Figure 1C). By comparison with the TE region of rice, the number of whole TE contained in RNA corresponding genes was very small (Figure 1D). It was speculated that many RNA corresponding DNA sequences overlap with TE region, but not completely contained. Slightly different from the results of previous studies, most lncRNAs and miRNAs corresponding genes contained no exons, except 57.14% circRNAs corresponding genes (Figure 1E). All mRNAs corresponding genes had exons, and most of them had fewer than 10 exons.

The transcription factor binding sites (TFBSs) of all detected ncRNA and mRNA corresponding genes loci were predicted by TSPTFBS (Liu et al., 2021). The results showed that TFBSs in mRNA corresponding genes loci were significantly more abundant than those in ncRNA corresponding genes loci. ANAC017 was the most common binding site of ncRNA corresponding genes loci, except for small RNAs identified in this study (Supplementary Figure 3). MiRNAs corresponding genes loci had significantly fewer binding sites than other ncRNAs.

Referring to the study of Crisp et al. (2020), the genome was divided into six meta-feature categories, including TE, noncoding RNA transcript loci, genic, intergenic, gene-proximal, and no-annotation. According to the ncRNA corresponding DNA sequences, the distribution of ncRNA corresponding genes in each genomic feature was determined (Supplementary Figure 4A). The number of lncRNAs corresponding DNA sequences overlapped with TE regions was higher than other regions. The corresponding DNA sequences of miRNAs (21, 22, and 24 nt) were mainly overlapped in TE, gene proximal and inter-gene regions. Further analysis found that the proportion of circRNAs corresponding DNA sequences located in exons was higher than that of other ncRNAs (Supplementary Figure 4B).

### Global ncRNA profiles of the hybrid and its parents

**Figure 2:**
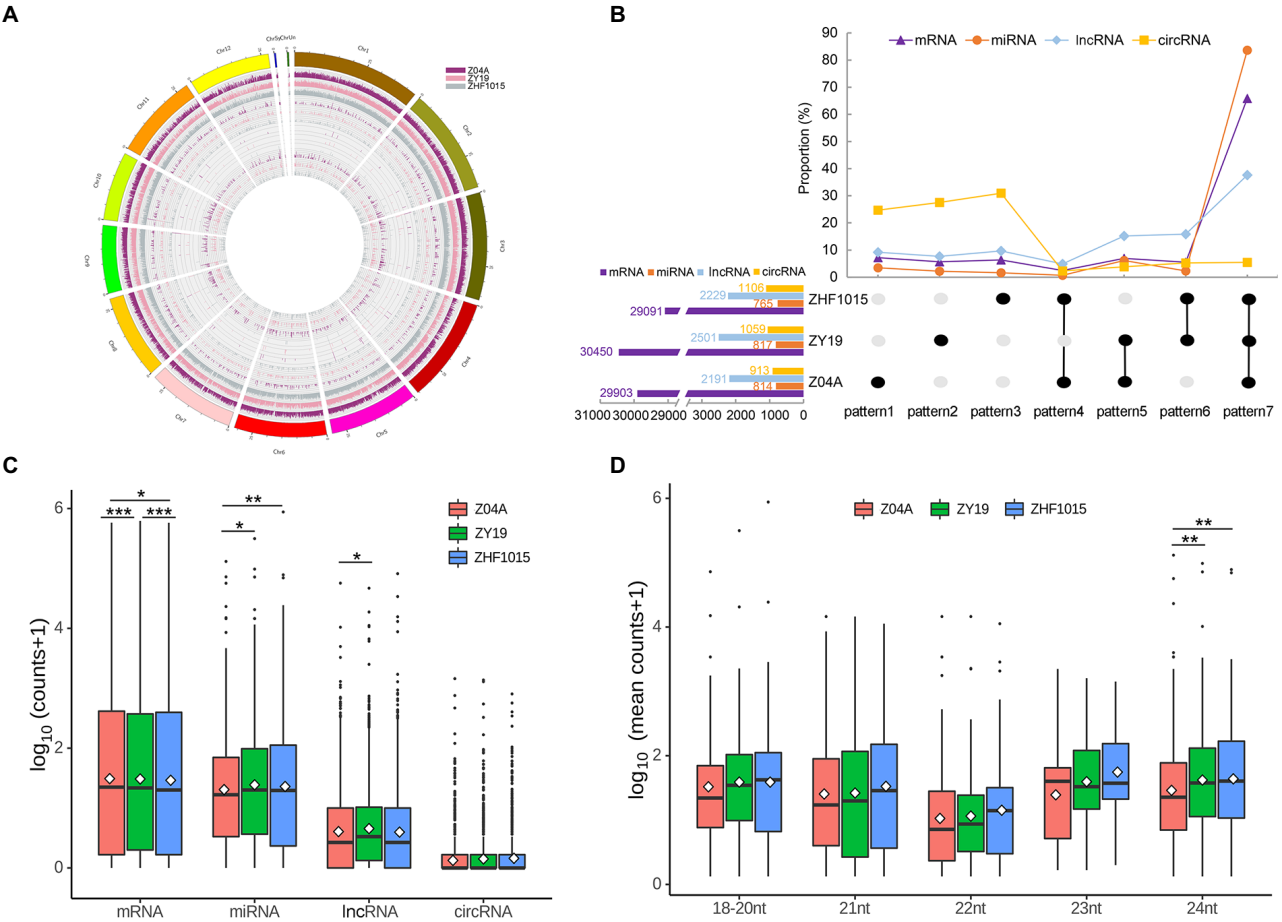
ncRNA profiles of the hybrid and its parents. **(A)** Location information and expression levels of four types of RNAs in the three varieties. mRNA, lncRNA, circRNA and miRNA from outside to inside. Different colors indicate different varieties. **(B)** The number of RNA expressed by different varieties. **(C)** Expression levels of four RNAs in the three varieties. **(D)** Expression levels of miRNA of different length in three varieties. *** means significant difference with *p* < 0.001, ** means significant difference with *p* < 0.01, and * means significant difference with *p* < 0.05.

After analyzing the sequence characteristics of all identified RNAs, the differences in ncRNA profiles between the hybrid and its parents were analyzed. Figure 2A shows the expression level of different types of RNA (mRNA, lncRNA, circRNA and miRNA from outside to inside) on different chromosomes of the three varieties, which showed that the expression of different types of RNAs was different.

Among the 867 identified miRNAs, 814, 817 and 765 miRNAs were expressed in maternal, hybrid and paternal lines, respectively (Figure 2B). The number of miRNAs expressed in all three varieties (co-expressed miRNA) accounted for the highest proportion, reaching 83.62%. The number of miRNAs expressed in both maternal and hybrid lines, but not expressed in paternal line, was the next (6.11%). Specifically expressed miRNAs in maternal (pattern 1), hybrid (pattern 2) and parental (pattern 3) lines were accounted for 3.46, 2.19 and 1.16% of the total identified miRNAs, respectively. Among 7 expression patterns of miRNAs, the number of pattern 4 (miRNAs that showed no expression in the hybrid line, but expressed in the maternal and paternal lines) was the least (Figure 2B).

Among the 3,278 lncRNAs identified in this study, 2,191, 2,501 and 2,229 lncRNAs were expressed in maternal, hybrid and paternal lines, respectively (Figure 2B). The result showed a larger number of lncRNAs in hybrid than that of its parents. The number of co-expressed lncRNAs was the highest (37.61%). Pattern 6 (lncRNAs expressed in both maternal and hybrid lines, but not in paternal lines) and Pattern 5 (lncRNAs expressed in both hybrid and paternal lines) were followed, accounting for 15.86 and 15.16%, respectively. The specific lncRNAs expressed in maternal line (Pattern 1), hybrid line (Pattern 2), and parental line (Pattern 3) accounted for 9.18, 7.66 and 9.64% of the total identified lncRNAs, respectively. Among the 7 lncRNA expression patterns, pattern 4 had the least amount (4.88%; Figure 2B).

In the total of 2,521 circRNAs, 913, 1,059 and 1,106 circRNAs were expressed in maternal, hybrid and paternal lines, respectively (Figure 2B). The proportion of circRNAs specifically expressed in three varieties (Pattern 1, Pattern 2, and Pattern 3) was higher than that of other patterns, 24.7, 27.56 and 30.94%, respectively. The pattern with the lowest proportion was Pattern 4, with just 2.3%.

As for mRNAs, 29,903, 30,450 and 29,091 transcripts were identified in the maternal, hybrid and paternal lines, respectively (Figure 2B). A total of 65.87% mRNA transcripts were expressed in all three varieties (Pattern 7). The proportion of mRNA with other expression patterns ranged from 2.46 to 7.19%, and pattern 4 had the lowest percentage (2.46%).

The proportion of co-expressed (pattern 7) miRNA was greater than that of mRNA and lncRNA. The proportion of co-expressed circRNAs was the lowest. Additionally, the number of expressed genes in the hybrid was much higher than that in the parents. Analyses of RNA expression patterns showed that mRNA, lncRNA and miRNA were relatively conserved, while the number of circRNAs with specific expression was higher in the three varieties.

The circRNA host genes and target genes of lncRNAs and miRNA were predicted (Supplementary Table 2). Based on the above analysis of RNA expression patterns, the function analysis of the corresponding target genes of seven expression patterns of RNA was carried out. Because the proportion of co-expressed (Pattern 7) miRNAs was the largest, the number of GO terms of target genes enriched was the largest (Supplementary Figure 5). Target genes of specifically expressed miRNAs in the maternal line and paternal line were significantly enriched in 7 and 4 GO items, respectively. In the hybrid, target genes of specific expression of miRNAs were mainly enriched in 5 GO items, including two biological processes (DNA-templated transcription, L-phenylalanine catabolic process) and molecular functions such as phenylalanine ammonialyase activity. There was no overlap among the GO items of the three specifically expressed miRNAs target genes. Interestingly, among the seven expression patterns of lncRNA, target genes of the 6 expression patterns except pattern 4 were significantly enriched in GO:0004190 (Supplementary Figure 6). GO:0004190 is a molecular function term related to aspartic-type endopeptidase activity. Specifically expressed lncRNAs in the hybrid were significantly enriched in GO:0046914 and GO:0016779, which are molecular functional items related to transition metal ion binding and nucleotidyltransferase activity, respectively. In three rice varieties, target genes of specifically expressed circRNA were enriched in the same GO items, such as GO:0009941, GO:0009570 and GO:0009535, which were related to the cell components of chloroplasts (Supplementary Figure 7).

To explore whether there were significant differences in the global expression of RNAs in the three varieties, the expression levels of four types of RNAs were analyzed. The results showed that there were significant differences in mRNA expression levels among different varieties (Figure 2C). The global expression level of miRNA was the highest among all ncRNAs, and it was at the level of high parental expression in the hybrid. The expression level of lncRNA in the hybrid was significantly higher than that in the maternal line, and there was no significant difference in the expression level between the hybrid and paternal lines. The global expression level of circRNA was the lowest among all ncRNAs, and there was no significant difference among the three varieties. MiRNAs of different lengths have various functions, so miRNAs were distinguished by length (Figure 2D). The results showed that most miRNAs except 18 to 20 nt miRNAs were at the mid-parent expression level. Among them, for only 24 nt miRNA, the global expression level in the hybrid was significantly different from that of the maternal line, and the global expression level of the hybrid was close to that of the paternal line.

### Differential expression ncRNAs (DEncRNAs) between hybrid and parents

**Figure 3:**
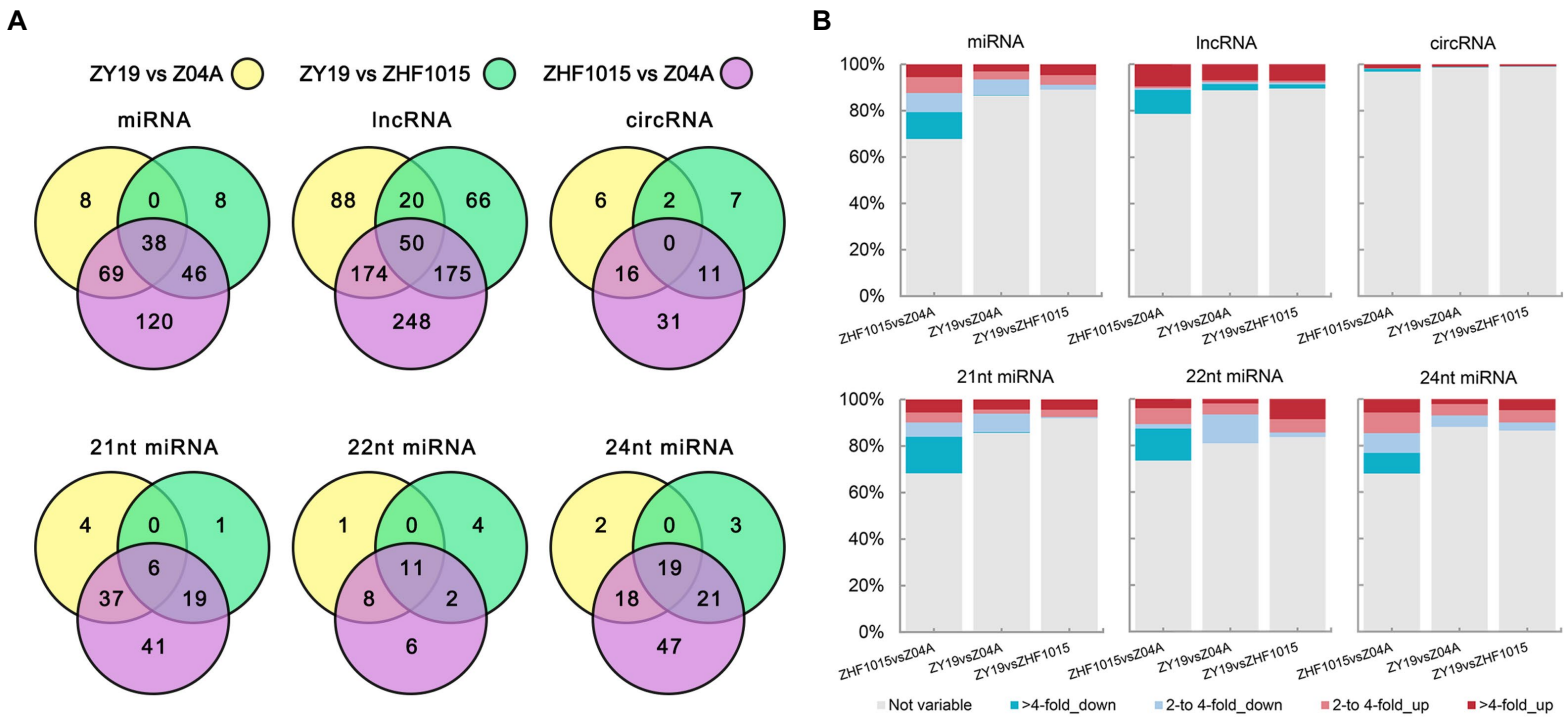
Differential expression ncRNAs between the three varieties. **(A)** Venn diagram of ncRNA of different difference groups. **(B)** Proportions of ncRNA with different levels of difference.

To identify heterosis-related ncRNAs in this hybrid rice combination, DEncRNAs were analyzed. The results indicated that the total number of DEncRNAs between the parents and hybrid was less than the number of DEncRNAs between the two parents for all types of ncRNA (Figure 3A). There were 179 DEmiRNA_HP_s identified and 237 corresponding DEmiRNA_PP_s. In DElncRNA identification, 573 DElncRNA_HP_s and 647 DElncRNA_PP_s were obtained. The number of DEcircRNA was the lowest, including 42 DEcircRNA_HP_s and 58 DEcircRNA_PP_s. Among the DEGs, the number of DEGs between the paternal line and hybrid (1305) was higher than that between the maternal line and hybrid (881; Supplementary Figure 8A). Except for 24 nt DEmiRNAs, the number of DEncRNAs between the hybrid and paternal lines was less than the number of DEncRNAs between the hybrid and maternal lines (Figure 3A). For example, there were 86 DEmiRNAs between the paternal line and hybrid and 115 DEmiRNAs between the maternal line and hybrid. In DElncRNA_HP_s, the number of DElncRNA_HP_s between the paternal line and hybrid was 311, and that between the maternal line and hybrid was 332.

In all ncRNAs identified, the proportion of DEmiRNA was higher (Figure 3B; Supplementary Figure 8B). This proportion was higher than that of DElncRNAs and DEcircRNAs. Moreover, compared with the fold change of DEncRNAs between the two parents, the fold change of DEncRNAs between the hybrid and parents was lower, which was especially evident in downregulated ncRNAs. After further studying the genetic pattern of DEncRNA_HP_s, it was found that the RNA expression level of the hybrid was influenced by the expression level of its parents (Supplementary Figure 8C). For example, most lncRNAs with higher expression levels in the hybrid than in the maternal line overlapped with DElnc_PP_RNAs, which showed high expression levels in the paternal line. This indicated that the high expression of these lncRNAs in the hybrid benefited from the high expression in the paternal line (Supplementary Figure 8C). Similarly, the low expression of ncRNA in the hybrid was also affected by the low expression of parental ncRNA. These results might be related to the formation of mid-parental expression and non-additive expression in the hybrid.

### Analysis of additive and non-additive expression of ncRNAs in hybrid

**Figure 4:**
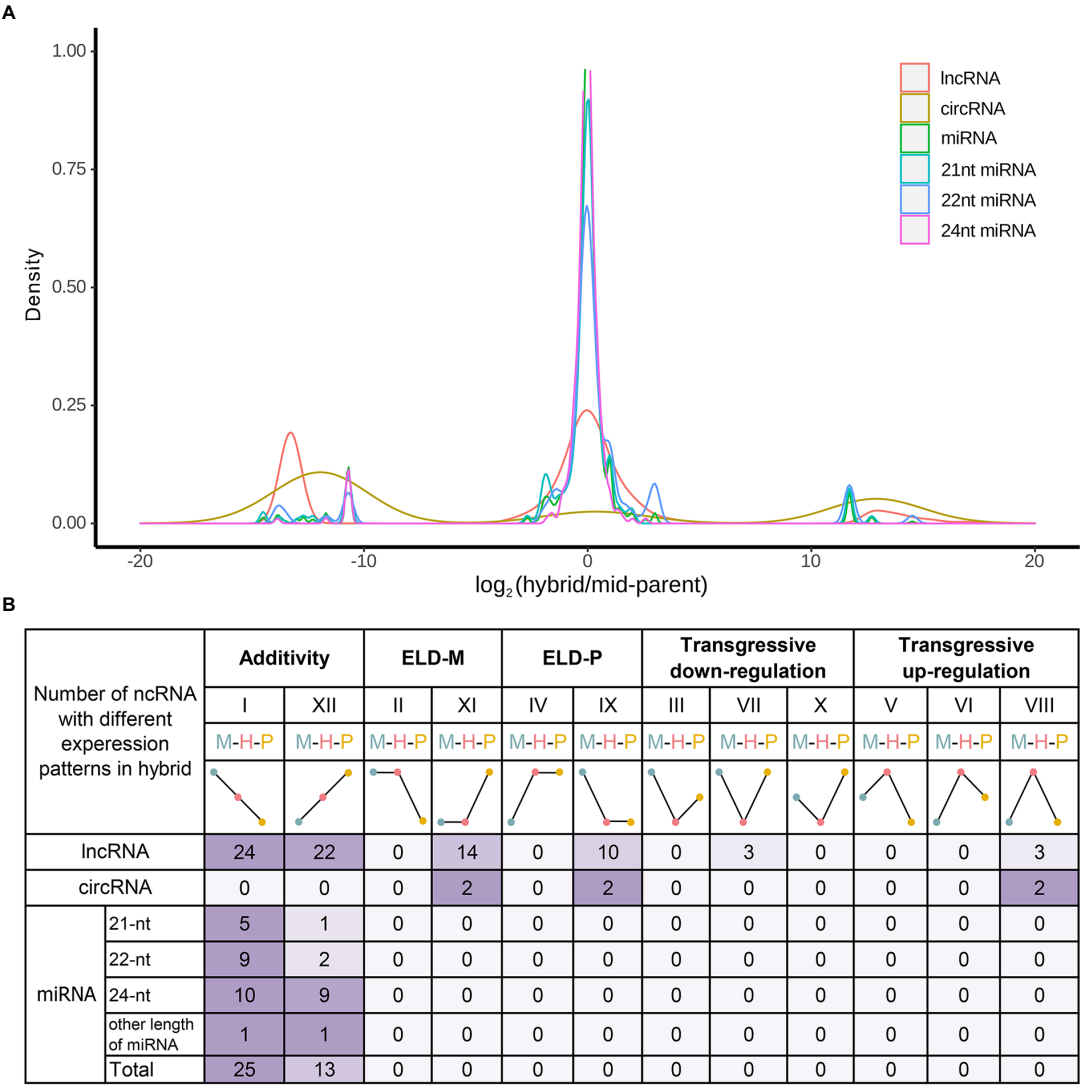
Additive and non-additive ncRNAs in hybrids. **(A)** Ratio distribution of hybrid and MPV in different ncRNAs. **(B)** Additive and non-additive patterns of ncRNAs. The 12 possible differential expression states in the F1 hybrid relative to its diploid parents. Roman numerals indicate the same categorization as used in Yoo et al. (2013).

Previous non-additive mRNA analysis showed that the number of additive genes in hybrid was more than that of non-additive, and there was a phenomenon of transgressive up/downregulated expression (Wang and Wang, 2022). Therefore, additive and non-additive analyses were performed on the identified ncRNAs in this study. Additive expression analysis showed that the expression level of most ncRNAs in hybrid was close to the mid-parent value except circRNA (Figure 4A). Assessed *via* Wald tests, ncRNAs in hybrid were divided into 12 expression patterns (Figure 4B). Except for circRNAs, the number of additively expressed ncRNAs was greater than that of non-additively expressed ncRNAs. Equivalent expression, that is, no change in ncRNA expression, accounted for the majority. Meanwhile, the above studies on the expression patterns and differential expression patterns of ncRNAs indicated that most ncRNA were conserved in hybrid and its parents. Therefore, it is necessary to further analyze the non-additive expression of ncRNAs in the hybrid.

**Figure 5:**
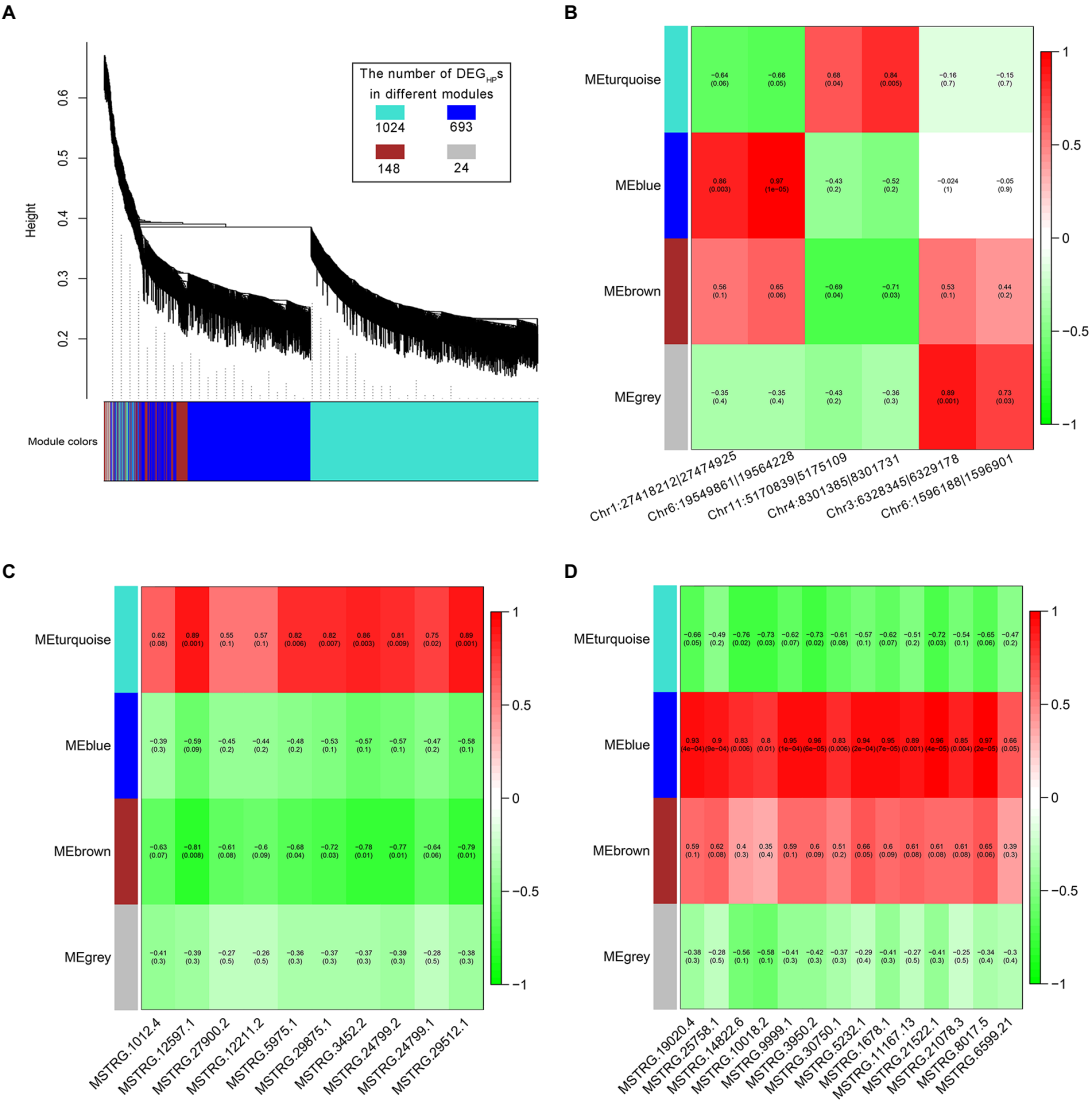
The regulation of non-additive expressed ncRNAs on DEGHPs. **(A)** Four expression patterns of DEGHPs obtained by WGCNA analysis. **(B)** Correlation analysis of non-additive expressed circRNAs and DEGHPs. **(C)** Correlation analysis of ELD-P expressed lncRNAs and DEGHPs. **(D)** Correlation analysis of ELD-M expressed lncRNAs and DEGHPs.

The differentially expressed genes between hybrid and parents (DEG_HP_s) have been found to be involved in the molecular mechanism of heterosis formation (Wei et al., 2009), so the correlation analysis between non-additive expressed ncRNAs and DEG_HP_s might be helpful to reveal the effect of ncRNAs on heterosis. WGCNA was used to analyze the regulation of non-additive expressed ncRNAs on DEG_HP_s. The results showed that DEG_HP_s could be divided into four expression patterns (Figure 5A). Among them, MEgrey represents the genes that were not clustered, and the number was only 24 (1.3% of the total DEG_HP_s). As for the 6 non-additive circRNAs (Figure 5B), 2 circRNAs (Chr1:27418212|27,474,925 and Chr6:19549861|19,564,228) were significantly positively correlated with the DEG_HP_s expression of MEblue, and Chr4:8301385|8,301,731 was significantly positively correlated with the DEG_HP_s expression of MEturquoise module (Figure 5B). Through correlation analysis, there was no significant correlation between the expression of 6 transgressive regulated lncRNA and DEG_HP_s in hybrid. Among the 10 lncRNAs in pattern ELD-P, 6 lncRNAs (MSTRG.12597.1, MSTRG.5975.1, MSTRG.29875.1, MSTRG.3452.2, MSTRG.24799.2 and MSTRG.29512.1) were significantly positively correlated with the expression of DEG_HP_s in MEturquoise module (Figure 5C). Meanwhile, MSTRG.12597.1 was significantly positively correlated with DEG_HP_s expression of MEturquoise module and negatively correlated with 148 MEbrown genes. Most of the lncRNAs in pattern ELD-M (13/14) were significantly positively correlated with the expression of 693 DEG_HP_s in MEblue module (Figure 5D).

### Heterosis-related lncRNA–mRNA and miRNA–mRNA networks

There are multilevel regulatory relationships between ncRNAs and mRNAs. MiRNAs directly regulate target mRNAs by binding them, and lncRNAs directly regulate mRNAs by *cis* and *trans* regulation. Differential expression of RNA between hybrid and parents was considered to be related to heterosis formation. To clarify the regulatory network of heterosis-related RNAs, the network of target genes of DEmiRNA_HP_s and DElncRNA_HP_s with differential expression between hybrid and its parents was analyzed (Supplementary Figure 9).

**Figure 6:**
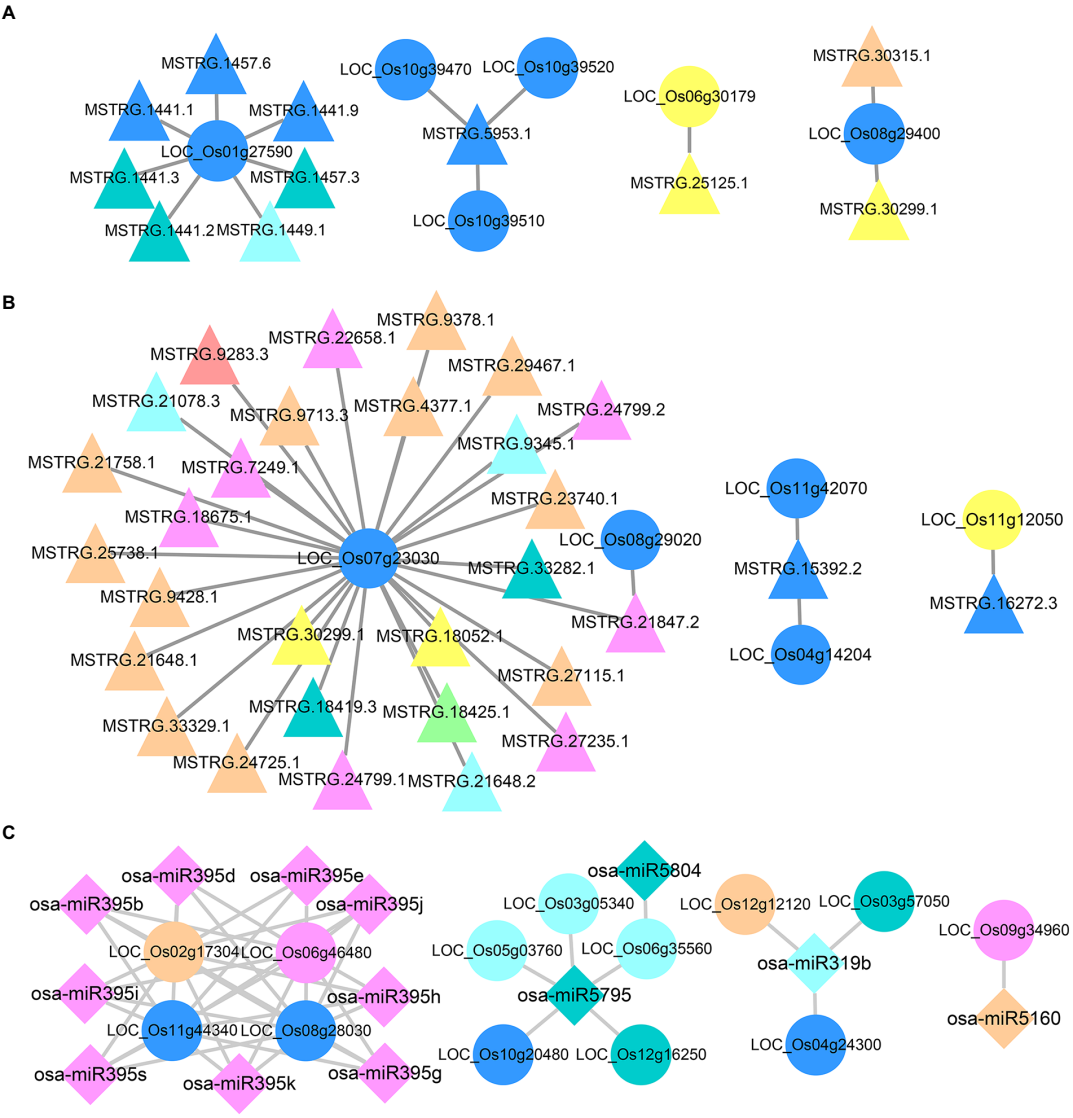
Regulatory networks of DElncRNAHPs and DEmiRNAHPs on their target DEGHPs. **(A)** Regulatory network of DElncRNAHPs on their cis target DEGHPs. **(B)** Regulatory network of DElncRNAHPs on their trans target DEGHPs. **(C)** Regulatory network of DEmiRNAHPs on their target DEGHPs. The shapes and colors of the different types of RNA were consistent with those illustrated in Supplementary Figure S9.

According to the expression patterns of DEncRNA_HP_s and DEG_HP_s in the hybrid, all DERNA_HP_s were divided into eight clusters. A total of 413 DEG_HP_s had *cis* targeting relationships with 357 DElncRNA_HP_s (Supplementary Figure 9A). The complex DElncRNA_HP_s-DEG_HP_s *cis* regulatory networks include one DEG_HP_ regulated by multiple DElncRNA_HP_s, multiple DEG_HP_s regulated by one DElncRNA_HP_s, and multiple DElncRNA_HP_s regulating multiple DEG_HP_s. LOC_Os01g27590, which was predicted to be a transposon protein, showed *cis* relationship with 7 lncRNAs (Figure 6A). Among them, the expression pattern of 3 lncRNAs in the hybrid was the same as that of LOC_Os01g27590, indicating that in hybrid the differential expression of LOC_Os01g27590 might be influenced by the expression of these three lncRNAs. MSTRG.5953.1 could *cis* target three DEG_HP_s simultaneously, and the differential expression level of these three DEGs in hybrids was the same as that of MSTRG.5953.1, which showed lower than that of the maternal line and higher than that of the paternal line (Figure 6A). LOC_Os06g30179, encoding cytochrome P450, was *cis*-targeted by MSTRG.25125.1 (Figure 6A). Both of them showed a pattern of higher expression in hybrid than in maternal line but lower expression than in paternal line. Therefore, it can be speculated that the expression of LOC_Os06g30179 may be related to the targeting of MSTRG.25125.1. At the same time, there were some DElncRNA_HP_s-DEG_HP_s networks with the opposite expression pattern to target genes in the hybrid. For example, LOC_Os06g29400 was targeted by both MSTRG.30315.1 and MSTRG.30299.1 (Figure 6A). The expression of MSTRG.30299.1 in the hybrid was higher than that in the maternal line, but lower than that in the paternal line. On the contrary, LOC_Os06g29400 expression level in hybrid was lower than that in maternal line and higher than that in paternal line. Similar opposing regulatory networks also deserve further investigation.

A total of 65 DElncRNA_HP_s and 34 DEG_HP_s had complex trans-targeting relationships (Supplementary Figure 9B). LOC_Os07g23030 was predicted to be a retrotransposon protein and was *trans*-regulated by 28 DElncRNA_HP_s, showed lower expression in the hybrid than in the maternal line, but higher than that in the paternal line (Figure 6B). MSTRG.30299.1 and MSTRG.18052.1 had the opposite expression pattern to LOC_Os07g23030 in hybrid, indicating that this gene might be indirectly involved in the heterosis-related lncRNA–mRNA network. LOC_Os11g12050, a disease resistance-related gene, was *trans* targeted by MSTRG.16272.3. A similar opposite expression pattern existed in the LOC_Os11g12050—MSTRG.16272.3 network (Figure 6B).

As shown in Supplementary Figure 9C, there were a total of 69 DEmiRNA_HP_s corresponding to 91 DEG_HP_s, and the relationship was complicated. A complex interaction network of multiple miRNAs targeting multiple identical mRNAs simultaneously emerged. In rice, osa-miR319b can negatively regulate tiller bud elongation, tiller number and yield per plant, which plays a critical role in leaf morphogenesis and organ boundary (Wang et al., 2021b). Osa-miR319b, as a DEmiRNA_HP_ in the hybrid, was involved in 3 DEG_HP_s regulatory networks (Figure 6C). Among them, 2 DEG_HP_s (LOC_Os04g24300 and LOC_Os12g12120) were potential disease resistance protein genes. LOC_Os03g57050 and osa-miR319b showed opposite expression patterns in hybrid. The expression level of LOC_Os03g57050 in the hybrid was higher than that in the paternal line, and the expression level of osa-miR319b in the hybrid was lower than that in the paternal. These results suggested that osa-miR319b might be involved in heterosis formation by miRNA-mRNA networks.

### Functional annotation analysis of heterosis-related lncRNA–mRNA and miRNA–mRNA network genes

To further investigate the potential role of lncRNA-mRNA and miRNA-mRNA networks in heterosis, the related target genes were annotated. *Cis* target DEG_HP_s of DElncRNA_HP_s were enriched to 9 and 11 terms in molecular function and biological process category, respectively (Supplementary Figure 10A). In molecular function, most *cis* target DEG_HP_s were labeled as ADP-binding and enzyme activity-related terms. Three *cis*-regulated DEG_HP_s were significantly enriched (*p* < 0.01) in terms related to sugar transmembrane transporter activity, and these three genes (LOC_Os05g12320, LOC_Os01g65880 and LOC_Os01g42110) all belonged to MtN3 family. The result indicated that these three genes and their corresponding DElncRNA_HP_s were involved in heterosis formation, and might have an effect on monosaccharide or polysaccharide transport in leaf of hybrid. In biological process, stress response terms such as defense response, immune response and response to bacterium were enriched. C*is* target DEG_HP_s were mainly enriched in KEGG pathways such as cyanoamino acid metabolism, nucleotide or base excision repair (Supplementary Figure 10B).

Different from *cis* target DEG_HP_s, *trans* target DEG_HP_s of DElncRNA_HP_s were enriched into fewer molecular function category than those in biological process (Supplementary Figure 11A). There were 11 and 9 GO terms in molecular function and biological process, respectively. Among them, 8 DEG_HP_s significantly enriched (*p* < 0.01) in defense response and response to UV were worthy of attention. KEGG analysis showed that only a few DEG_HP_s were enriched, such as other glycan degradation and amino acid metabolism pathways (Supplementary Figure 11B).

Target DEG_HP_s of DEmiRNA_HP_s were enriched to 10 molecular function, 8 biological process and 2 cellular component categories (Supplementary Figure 12A). In molecular function, most DEG_HP_s were labeled to DNA binding and enzyme activity-related terms. Target DEG_HP_s were enriched in biosynthetic metabolism of KEGG analysis showed that these heterosis-related DEmiRNA_HP_s-DEG_HP_s networks were mainly enriched in metabolic pathways (Supplementary Figure 12B).

### Interaction network analysis of ASEGs with lncRNA and miRNA in the hybrid

ASEG analysis has been shown to be associated with heterosis, but there are few studies on whether these genes are regulated by ncRNAs (Lv et al., 2019). Analysis of ASEGs previously identified in ZY19 (Wang and Wang, 2022) showed that 775 maternal ASEGs were *cis*-regulated by 1,294 lncRNAs, showing 2,116 interaction pairs (Supplementary Figure 13). These interaction networks included both complex relationship pairs, such as an ASEG (LOC_Os07g28040, presumably an o-methyltransferase gene) regulated by multiple lncRNAs, and many one-to-one maternal ASEG-lncRNAs relationship pairs. A total of 205 maternal ASEGs were *trans*-regulated by 207 lncRNAs, presenting 208 pairs of interaction (Supplementary Figure 14). Compared with *cis* ASEG-lncRNA network, the number of *trans* ASEG-lncRNAs network was small and the interaction relationship was simple. In ZY19, 512 paternal ASEGs were *cis*-regulated by 979 lncRNAs, showing 1,382 pairs of interaction (Supplementary Figure 15). A total of 124 genes with paternal expression bias were *trans*-regulated by 146 lncRNAs, showing 167 pairs of interaction (Supplementary Figure 16). The interaction network between miRNA and ASEG was more complex, and multiple miRNAs participate in the expression of multiple ASEG simultaneously. A total of 379 maternal ASEGs and 386 miRNAs presented 986 pairs of relationship, and 257 paternal ASEGs and 341 miRNAs presented 684 pairs of relationship (Supplementary Figure S17, S18).

### circRNA–host gene network analysis and its role in heterosis

**Figure 7:**
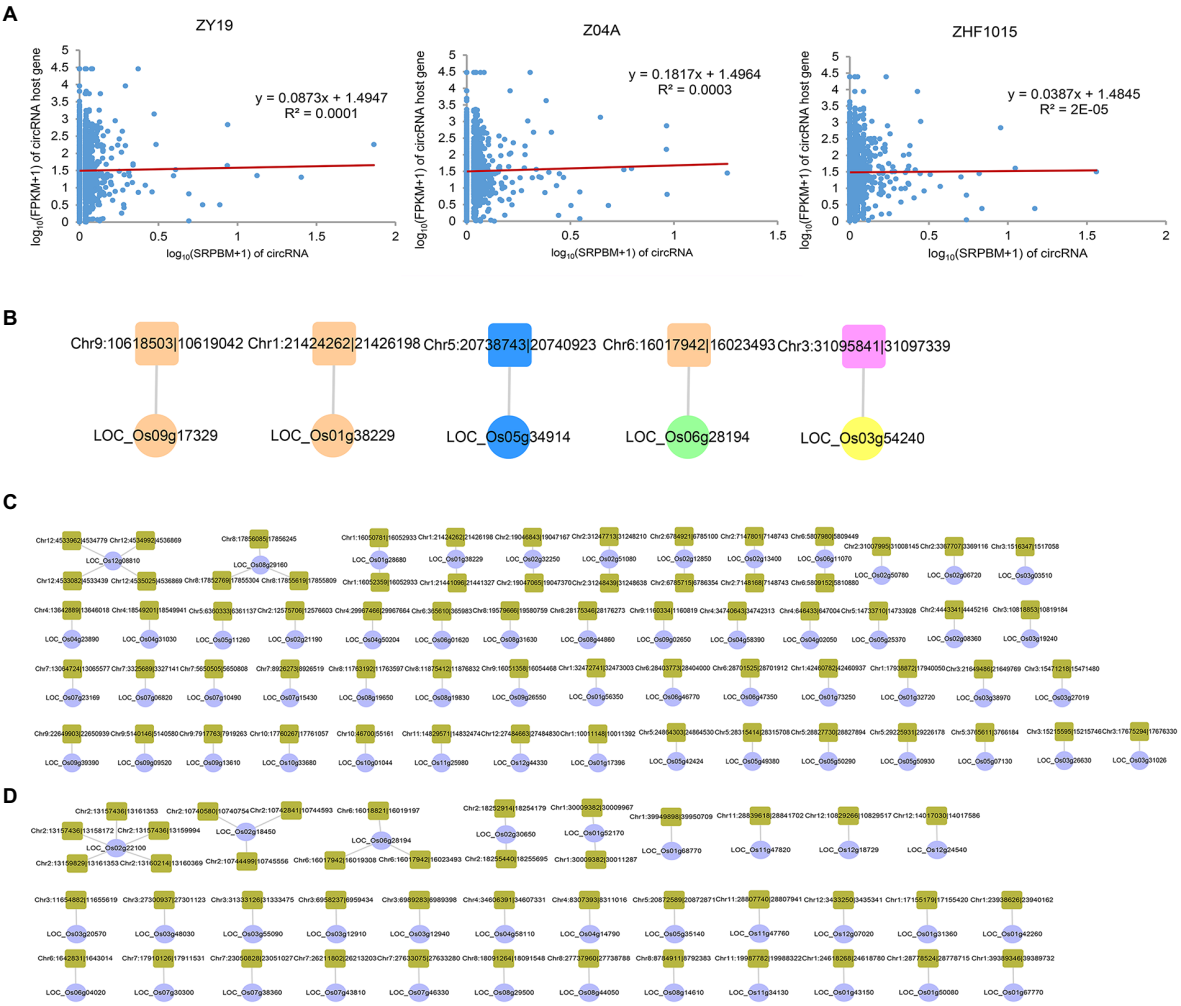
Regulatory networks of circRNAs and their host genes. **(A)** Correlation analysis of circRNAs and their host genes. **(B)** Regulatory network of DEcircRNAHPs and their host DEGHPs. The host DEGHPs and DEcircRNAHPs were represented by circle and square. The colors of the different types of RNA were consistent with those illustrated in Supplementary Figure S9. **(C)** circRNA-host ASEG-M network. The host ASEG-M and circRNA were represented by circle and square. **(D)** circRNA-host ASEG-P network. The host ASEG-P and circRNA were represented by circle and square.

Exploring the potential relationship between circRNAs and their host genes is necessary for further study of circRNAs. In order to understand whether circRNA was related to the expression level of host gene, correlation analysis was conducted on the expression level of circRNA (SRPBM) and its parent gene (FPKM) in the three varieties (Figure 7A). The results showed that there was a weak positive correlation between the expression level of circRNA and its host gene, which was consistent with previous research (Zhou et al., 2021).

To clarify potential circRNA–host gene network involved in the formation of heterosis, DEcircRNA_HP_s and its corresponding host DEG_HP_s were analyzed (Figure 7B). Compared with miRNA and lncRNA, the networks of DEcircRNA_HP_s-host DEG_HP_s were simple. In 3 groups of DEcircRNA_HP_s-host DEG_HP_s interaction networks, the expression patterns of DEcircRNA_HP_s and its host DEG_HP_s in hybrid were the same, which showed that the three circRNA (Chr1:21424262|21,426,198, Chr5:20738743|20,740,923, Chr9:10618503|10,619,042) and their host genes might be involved in inter-subspecific hybrid heterosis. This result might be important for exploring the function and origin of circRNA in heterosis.

As mentioned above, the research on ASEG is of great help in understanding the molecular mechanism of heterosis. Therefore, the regulatory network of host ASEGs and circRNAs in hybrid was worth studying. Among the maternal ASEGs of hybrid, there were 55 host genes corresponding to 67 circRNAs, forming 67 pairs of circRNA–host ASEG network (Figure 7C). Most of them were one-to-one simple networks, but there were also complex networks, such as the network with LOC_Os12g08810 as the core host ASEG, which is also called *OsGGP*, encodes galactose phosphorylase and is crucial for plant stress resistance and development. It is the host gene of four circRNAs (Chr12:4533082|4,533,439, Chr12:4533962|4,534,779, Chr12:4534992|4,536,869, Chr12:4535025|4,536,869). A total of 33 paternal host ASEGs were identified, corresponding to 43 circRNAs, forming 43 pairs of circRNA–host ASEG network (Figure 7D). In this network, the most complex one consisted of 5 circRNAs and 1 host ASEG-P (*RHMBD6*).

### Heterosis-related miRNA–RNA network and heterosis-associated ceRNA network construction

Some lncRNAs and circRNAs can act as ceRNAs and competitively bind miRNAs, thus affecting the regulation of miRNAs on target mRNAs. Through TargetFinder analysis, 4,693 pairs of miRNA–lncRNA interaction and 6,258 pairs of miRNA–circRNA interaction were obtained among the identified ncRNAs, including 592 miRNAs, 1,546 lncRNAs and 859 circRNAs (Supplementary Table 3). Heterosis-related ceRNA regulatory networks were established by combining miRNA target genes with DEncRNA_HP_s in the hybrid.

**Figure 8:**
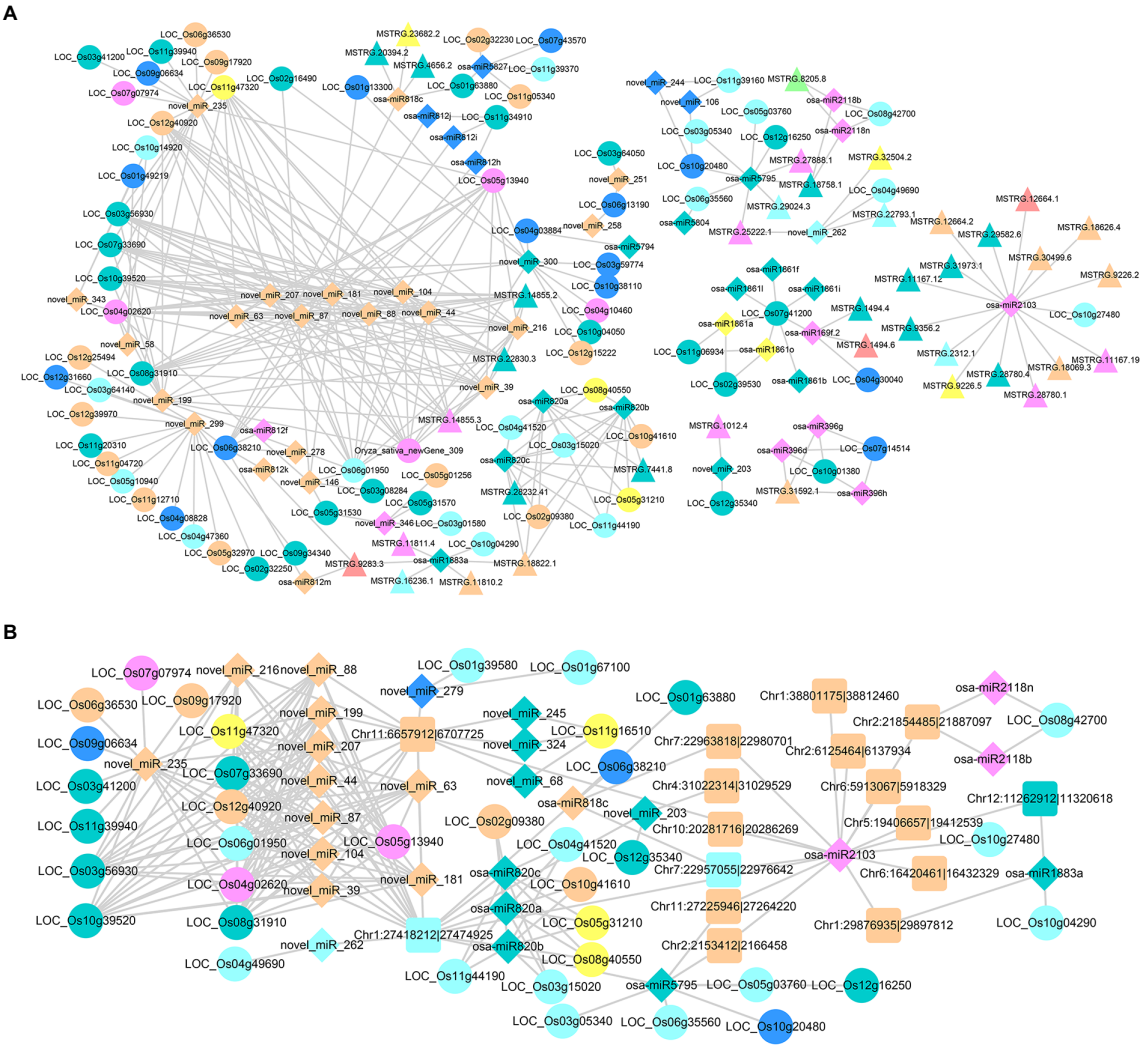
Regulatory networks of DEmiRNAHPs and ceRNAs. **(A)** Regulatory network of DElncRNAHPs as ceRNA. **(B)** Regulatory network of DEcircRNAHPs as ceRNA. The target DEGHPs, DElncRNAHPs, DEmiRNAHPs and DEcircRNAHPs were represented by circle, triangle, diamond and square, respectively. The colors of the different types of RNA were consistent with those illustrated in Supplementary Figure S9.

In the construction of DElncRNA_HP_s-DEmiRNA_HP_s-DEG_HP_s axes, we obtained complex ceRNA networks, and most of them were centered on miRNA (Figure 8A). There were 52 miRNAs, 39 lncRNAs and 77 DEG_HP_s, forming 6 groups of ceRNA interaction networks. In other words, 39 lncRNAs related to heterosis were considered as potential ceRNAs to influence the regulation of miRNAs on their target genes. Based on targeting relationships among different networks, complex ceRNA networks were formed (Figure 8A). As shown in Figure 8A, 15 DElncRNA_HP_s simultaneously acted as ceRNAs of osa-miR2103, affecting its regulation of LOC_Os10g27480. LOC_Os10g27480 is located on chromosome 10 of rice and its current function has not been studied. These 17 RNAs were differentially expressed in the hybrid, and also identified as potential ceRNAs. Further study of this network may contribute to a better understanding the contribution of ceRNA to heterosis formation. At the same time, there were also some complex networks of multiple DElncRNA_HP_s acting as ceRNAs of multiple DEmiRNA_HP_s (including some novel miRNAs identified in this study, such as novel_miR_207, novel_miR_181 and so on). For example, the miR2118 family to which osa-miR2118b belongs is a relatively conserved 22 nt miRNA superfamily in plants, which plays important role in plant growth and development, disease resistance and resistance (Zhang et al., 2022b). Osa-miR2118b was differentially expressed in hybrid and its parents, and two lncRNAs (MSTRG.8205.8 and MSTRG.18758.1) were acted as ceRNAs of osa-miR2118b, affecting the regulation of osa-miR2118b on LOC_Os08g42700 (Figure 8A). LOC_Os08g42700 is presumed to be a resistance protein gene. This is also a set of ceRNA network with potential heterosis that deserve further study.

There were 26 miRNAs, 16 circRNAs and 37 DEG_HP_s in the DEcircRNA_HP_s-DEmiRNA_HP_s-DEG_HP_s interaction network, forming one complex ceRNA interaction network (Figure 8B). The results showed that Chr2:21854485|21,887,097 was the ceRNA of osa-miR2118n and osa-miR2118b, and influence the regulation of these two miRNAs on LOC_Os08g42700 (Figure 8B). Both osa-miR2118n and osa-miR2118b belong to the miR2118 family, which has been mentioned above. At the same time, Chr1:27418212|27,474,925 can competitively bind with osa-miR820a，osa-miR820b and osa-miR820c, thus affecting the regulation of these three miRNAs on target genes. MiR820 is related to methyltransferase synthesis under pest stress, which affects rice growth and development (Tang and Chu, 2017). Most of the target genes corresponding to osa-miR820a，osa-miR820b and osa-miR820c were speculated to be related to retrotransposon protein-coding genes. These results suggest that these ceRNAs are not only involved in the formation of heterosis, but also have important functions in rice.

## Discussion

The molecular mechanism of heterosis is still unclear through extensive studies on genome, transcriptome and epigenetics (Larièpe et al., 2012; Shen et al., 2014; Shao et al., 2019; Wang and Wang, 2022; Zhang, 2022). In recent years, several ncRNAs and their corresponding regulatory networks have been proved to play important roles in plant growth and development (Ohtani, 2017; Yuan et al., 2018; Luan et al., 2019; Wang et al., 2020; Zhai et al., 2020; Chand Jha et al., 2021; Qiao et al., 2021). However, there are few studies on the role of ncRNA in plant heterosis so far, which have been conducted in maize and cabbage (Crisp et al., 2020; Hou et al., 2020; Li et al., 2021). As a hybrid rice with high yield potential, *indica* × *japonica* hybrid rice has always been an important material for heterosis research (Xiong et al., 2021; Zhang, 2022). This study demonstrated that ncRNA plays an important role in the heterosis of inter-subspecific hybrid rice by comprehensively analyzing the expression profile of ncRNA and the ncRNA regulatory network related to heterosis.

### Specific expression of different types of ncRNA in hybrid

In recent years, studies on plant ncRNAs have mainly focused on lncRNAs, circRNAs and miRNAs (Dou et al., 2017; Wang et al., 2018, 2021c; Li et al., 2020; You et al., 2021; Zhou et al., 2021). Due to the differences in the sources and functions of these three types of ncRNAs, they play a role in various plant growth and development processes (Voinnet, 2009; Tang and Chu, 2017; Zhai et al., 2020; Chand Jha et al., 2021). A total of 3,278 lncRNAs, 2,521 circRNAs and 867 miRNAs (including 345 newly predicted miRNAs) were identified in this study. Different types of ncRNAs corresponded to different length of DNA sequences, which might result in different ORF length, TE numbers and exon numbers of lncRNA, circRNA and miRNA corresponding genes. The majority of lncRNA encoded DNA sequences (99.27%) contained 0 to 100 nt ORFs. This result was consistent with the DNA sequence characteristics of corresponding to lncRNA identified in young panicles of rice (Wang et al., 2021c). At the same time, our study founded that a small number of circRNAs (10.24%) corresponding DNA sequences contained ORFs, while miRNA corresponding genes did not contain ORFs. TFBS prediction results showed that the DNA sequences corresponding to circRNAs had more TFBS than the other two ncRNAs (lncRNA and miRNA). ANAC017 is an endoplasmic reticulum binding transcription factor involved in the response of Arabidopsis cell wall to aluminum stress (Tao et al., 2022). Except for miRNA, ANAC017 was the most common predicted binding site for the other two ncRNA (lncRNA and circRNA) corresponding genes. TFBS prediction of miRNA showed only 4 different transcription factors could bind to miRNA corresponding genes. These results will provide clues for the study of lncRNA, circRNA and miRNA in rice.

Previous studies have shown that hybridization can activate or inhibit gene expression in hybrids (Wei et al., 2009; Lv et al., 2019). In this study, we explored the effect of hybridization on ncRNA expression profiles of hybrid and its parents. The results showed that the expression numbers of lncRNA and miRNA in hybrid were higher than those in parents except for circRNAs. And the hybrid-specific expression of ncRNA (pattern 2) may provide valuable clues to the formation of heterosis. Among these ncRNAs, the specific expression of circRNAs was evident. Then, by GO functional annotation of genes targeted by these specific expression ncRNAs, most of them were related to chlorophyll biosynthesis. These results suggested that these specific expression ncRNAs in the hybrid might be related to flag leaf heterosis of inter-subspecific hybrid rice.

In maize, studies on heterosis-related small RNA of different lengths showed that there were different expression patterns of 21, 22, 23 nt small RNA in different maize samples, and overdominance expression of small RNA were rarely occurred in hybrids. In this study, results indicated significant differences between hybrid and parental miRNA profiles. The expression level of 21, 22, 23 nt miRNA in the hybrid showed no difference with the parents, but the expression level of 24 nt miRNA in the hybrid was significantly different with its parents (Figure 2D). This indicated that miRNAs with different functions have different contributions to genetics, suggesting that we need to conduct further analysis of miRNAs with different lengths.

### The important role of lncRNA in inter-subspecific hybrid rice heterosis formation

In plant, lncRNAs play key roles in development, reproduction, responses to light, stress responses and many other processes (Wang et al., 2014, 2020; Zhang et al., 2014; Jha et al., 2020). The expression of lncRNAs in rice shows tissue-specific and stage-specific (Zhang et al., 2014). An endogenous lncRNA, TWISTED LEAF (TL), plays a *cis*-regulatory role on *OsMYB60* in leaf morphological development (Liu et al., 2018). For anther-development, LDMAR regulates photoperiod-sensitive male sterility, an essential component of hybrid rice, and the rice epiallele *Epi-*sp., which encodes an lncRNA, was found to affect panicle architecture (Ding et al., 2012; Luan et al., 2019). The overexpression of lncRNA *LAIR* increases rice grain yield and upregulates some neighboring gene expression (Wang et al., 2018). Parental expression level dominance lncRNAs were identified in rice backcross introgression lines (Li et al., 2020), but lncRNA expression profile and the important role of lncRNA in heterosis formation of inter-subspecific hybrid rice need to be studied.

In this study, lncRNA expression profile showed variety-specific expression. At the same time, the DElncRNA_HP_s-DEG_HP_s *cis*/*trans* regulatory network related to heterosis was obtained in the inter-subspecific hybrid rice combinations (Supplementary Figures 9A,B). *Cis* target gene LOC_Os01g27590 corresponding to the 7 lncRNAs merit further research, especially MSTRG.1441.1, MSTRG.1457.6 and MSTRG.1441.9. The differential expression pattern of LOC_Os01g27590 in hybrid relative to its parents was the same as these three lncRNAs above, indicating that the expression of LOC_Os01g27590 in hybrid was related to these three lncRNAs, which may affect the formation of heterosis in hybrid. DElncRNA_HP_s-DEG_HP_s networks with opposite expression pattern were also worthy of further study. For example, the network of MSTRG.30315.1/MSTRG.30299.1 and their *cis* target LOC_Os06g29400. The number of *trans* regulatory networks of lncRNA is small, but they also provide important clues for heterosis-related networks. LOC_Os07g23030 and its corresponding 28 DElncRNA_HP_s formed a network that indirectly affected heterosis. In this network, although LOC_Os07g23030 was differentially expressed in hybrid relative to its parents, its corresponding DElncRNA_HP_s differential expression pattern was different. In addition to the above-mentioned multiple lncRNA *trans*-targeting the same gene, MSTRG.15392.2 can simultaneously target two DEG_HP_s and the differential expression pattern of these two genes (LOC_Os11g42070 and LOC_Os04g14204) showed the same with MSTRG.15392.2 in hybrid. These DElncRNA_HP_s-DEG_HP_s networks might play an important role in heterosis formation.

### The potential role of circRNAs in the heterosis

Among the circRNAs identified by Dou et al. (2017) in leaves of *Arabidopsis*, a certain number of circRNA related genes were enriched in the photosynthetic system. Meanwhile, circRNAs are involved in the response of Arabidopsis to heat stress (Pan et al., 2018). In wheat leaves, 88 candidate circRNAs were identified under dehydration stress, revealing a possible relationship between circRNAs and leaf dehydration response (Wang et al., 2017). Studies on the circRNAs expression profile of maize showed that circRNAs were involved in the response of stress pathway, and their expression diversity was affected by genetic background, which indicated that circRNAs may be involved in various regulatory networks in plant (Han et al., 2020). As for rice, it is necessary to study how circRNAs are affected by the genetic differences between *indica* and *japonica* and their roles in the formation of inter-subspecific hybrid rice. In the three varieties in this study, circRNAs were expressed at low levels and with high specificity. A total of five heterosis-related circRNAs and their host gene networks were obtained, among which three groups were highly correlated with heterosis. It is speculated that the differential expression of host gene in hybrid affects the corresponding circRNAs. The interaction analysis between ASEG and circRNAs is helpful for further study of the molecular mechanism of heterosis. In this study, 67 pairs of circRNA-host ASEG networks were obtained, which provided clues for further research on the influence of circRNA in the hybrid on allele-specific expression.

### Important contribution of miRNAs and their ceRNAs to heterosis formation

In the past few years, several research groups have reported that some miRNAs are involved in regulating rice growth, development and the morphogenesis of shoot architecture (Tang and Chu, 2017; Qiao et al., 2021). *OsASI1* is involved in the processing of a miRNA precursor and regulates the abundance of miRNA, which is essential for rice pollen development and flowering (You et al., 2021). Rice miRNAs can coordinate both immune and agronomic traits. MiR168 has been identified to be related to immune response in rice (Wu et al., 2015). A recent study showed that overexpression of miR168 significantly inhibited the expression of *AGO1*, resulting in increased sensitivity of rice to *Magnaporthe oryzae*, delayed growth period, and decreased yield-related traits (Wang et al., 2021a). In this study, osa-miR168a-3p and osa-miR168a-5p were highly expressed in hybrid and its parents. Osa-miR535 positively regulates tillering and negatively regulates yield and disease resistance by targeting *OsSPL14* (Zhang et al., 2022a). In this study, osa-miR535-5p was highly expressed in the maternal line, but at a low expression level in both the hybrid and the paternal line. It might be that low expression in the paternal line affected the expression level of osa-miR535-5p in the hybrid.

Analysis of miRNA expression profiles in maize and Chinese cabbage suggested the potential contribution of miRNA-mediated regulatory networks to heterosis (Hou et al., 2020; Li et al., 2021). Regrettably, there are few studies on the molecular mechanism of miRNA-mediated regulatory networks and rice heterosis. In the DElncRNA_HP_s-DEmiRNA_HP_s-DEG_HP_s and DEcircRNA_HP_s-DEmiRNA_HP_s-DEG_HP_s networks obtained in this study, 39 and 16 ceRNAs (lncRNA and circRNA) were predicted, respectively. Among them, MSTRG.8205.8, MSTRG.18758.1 and Chr1:27418212|27,474,925 was forecast for ceRNA associated with heterosis. At the same time, osa-miR820c was proved to be involved not only in the resistance response of rice (Tang and Chu, 2017), but also in the ceRNA network of heterosis formation. Members of osa-miR820 and osa-miR2118 families played important roles in these ceRNA networks, and the results would provide clues for subsequent studies on ceRNA networks and heterosis.

In summary, 867 miRNAs, 3,278 lncRNAs and 2,521 circRNAs were identified in the inter-subspecific hybrid rice combination. Some DElncRNA_HP_s-DEG_HP_s, DEmiRNA_HP_s-DEG_HP_s and circRNA-host gene interaction networks were obtained by analyzing the differentially expressed RNAs between hybrid and its parents. For example, the network of seven DElncRNA_HP_s *cis* regulated LOC_Os01g27590, and the network of 28 DElncRNA_HP_s simultaneously *trans* regulated LOC_Os07g23030. The DEG_HP_s regulatory networks involved in osa-miR319b, which plays an important role in rice leaves, were also analyzed. This study demonstrated multiple ncRNA regulatory networks in which ASEG participated. These might provide reference networks for further study of the effects of ncRNAs (miRNA, lncRNA, and circRNA) on heterosis. At the same time, we obtained two groups of complex DElncRNA_HP_s and DEcircRNA_HP_s as ceRNA to involved in the regulatory network of miRNA on target genes. These results not only indicated that the expression plasticity of ncRNAs was important for the heterosis of inter-subspecific hybrid rice, but also provides clues for the further analysis of ncRNA-heterosis-related gene networks.

## Data availability statement

Our data have been uploaded to NCBI Sequence Read Archive (SRA) database with accession number SRR19994724-41 (PRJNA855983).

## Author contributions

MW performed material preparation, data analysis, and manuscript writing. JW designed the project and modified the manuscript. All authors contributed to the article and approved the submitted version.

## Funding

This work was supported by the State Key Basic Research and Development Plan of China (2013CB126900).

## Conflict of interest

The authors declare that the research was conducted in the absence of any commercial or financial relationships that could be construed as a potential conflict of interest.

## Publisher’s note

All claims expressed in this article are solely those of the authors and do not necessarily represent those of their affiliated organizations, or those of the publisher, the editors and the reviewers. Any product that may be evaluated in this article, or claim that may be made by its manufacturer, is not guaranteed or endorsed by the publisher.

## Footnotes

^1^ http://rice.plantbiology.msu.edu/
