# Supplementary figures and images for: Non-coding RNA expression analysis revealed the molecular mechanism of flag leaf heterosis in inter-subspecific hybrid rice
Source: Front Plant Sci. 2022 Sep 26;13:990656. doi: 10.3389/fpls.2022.990656 (PMC9549252; doi:10.3389/fpls.2022.990656)

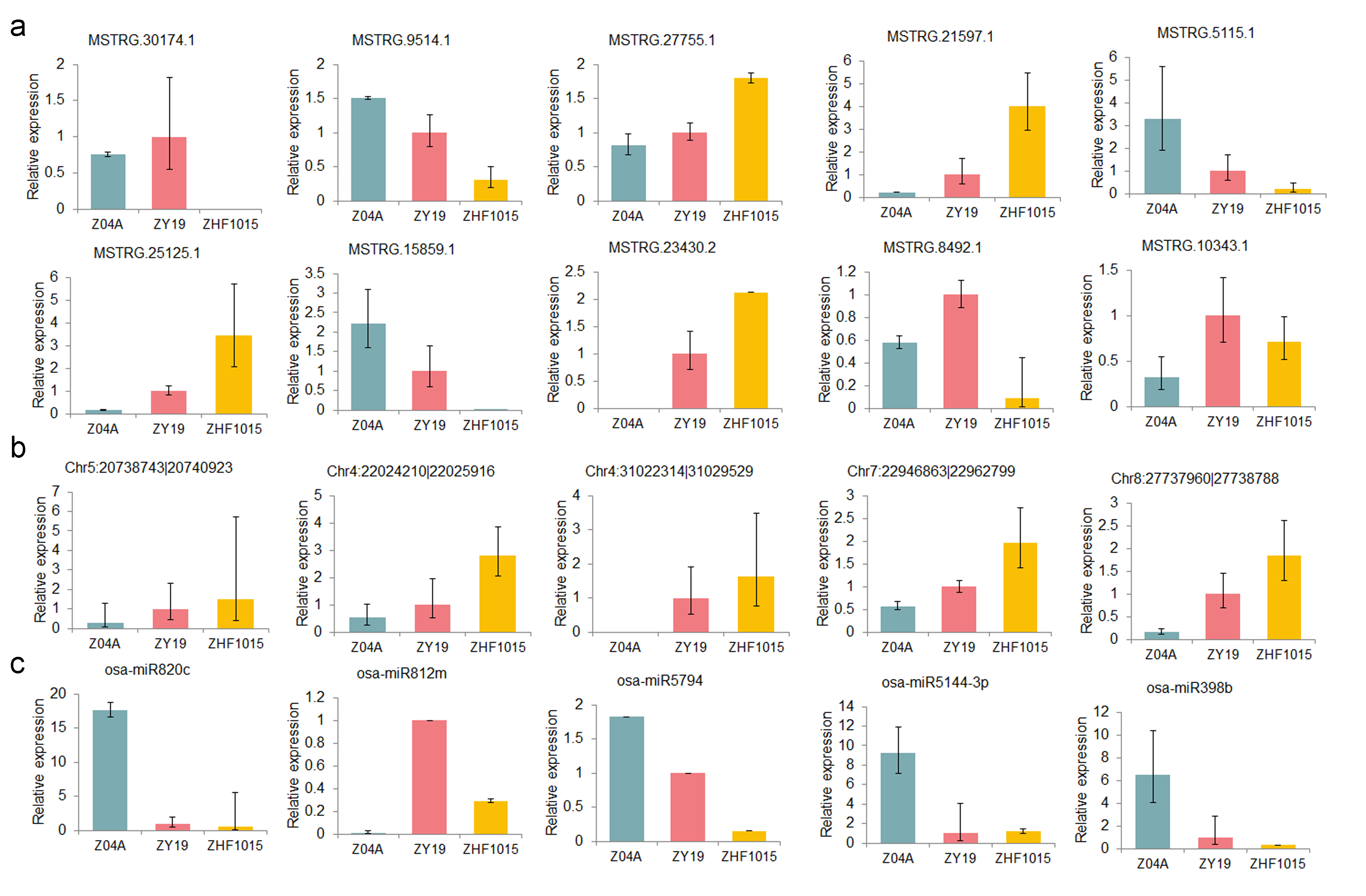

Supplement: Supplementary file 4 [file Data_Sheet_1.zip › Image 1.TIF]

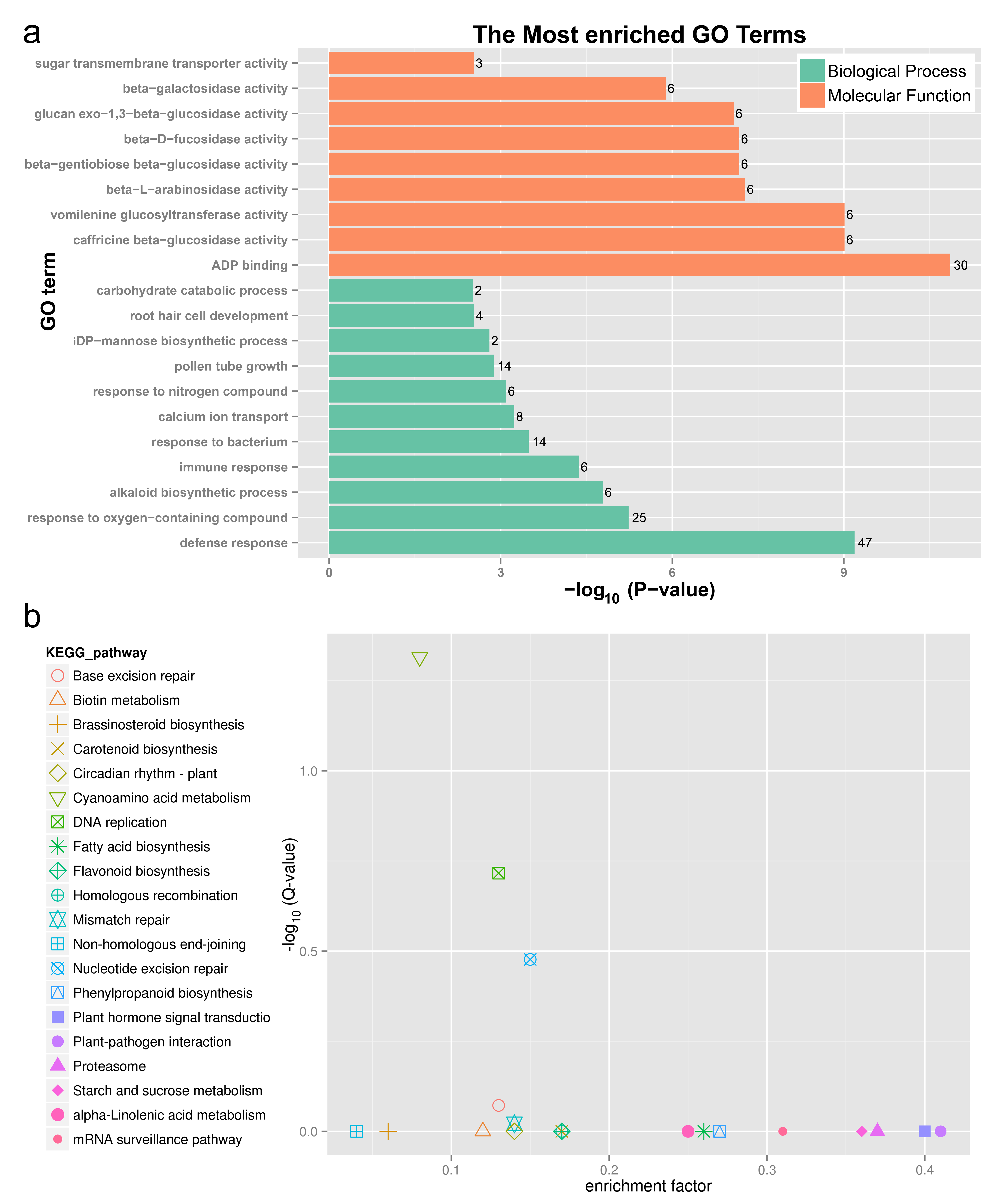

Supplement: Supplementary file 4 [file Data_Sheet_1.zip › Image 10.TIF]

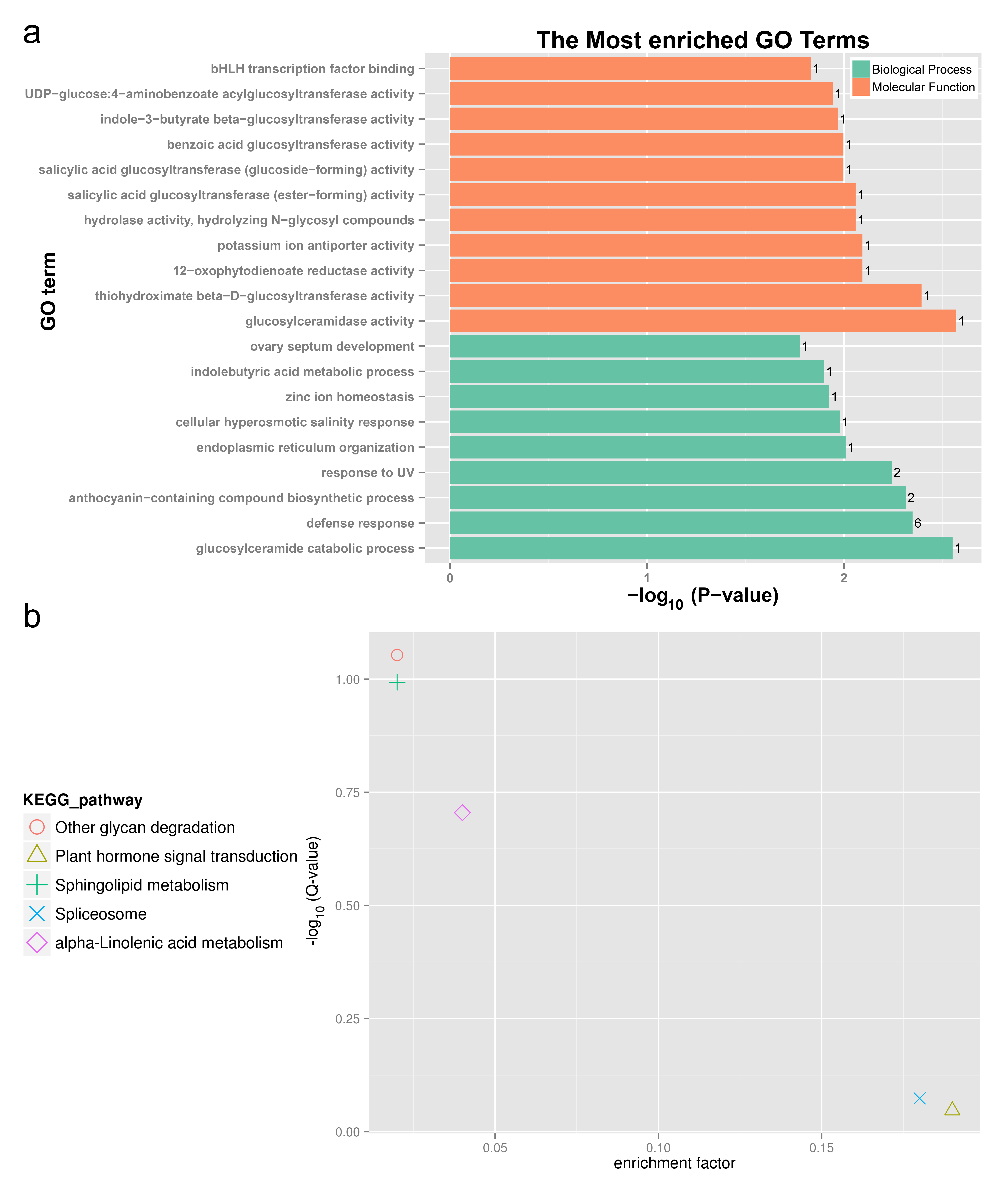

Supplement: Supplementary file 4 [file Data_Sheet_1.zip › Image 11.TIF]

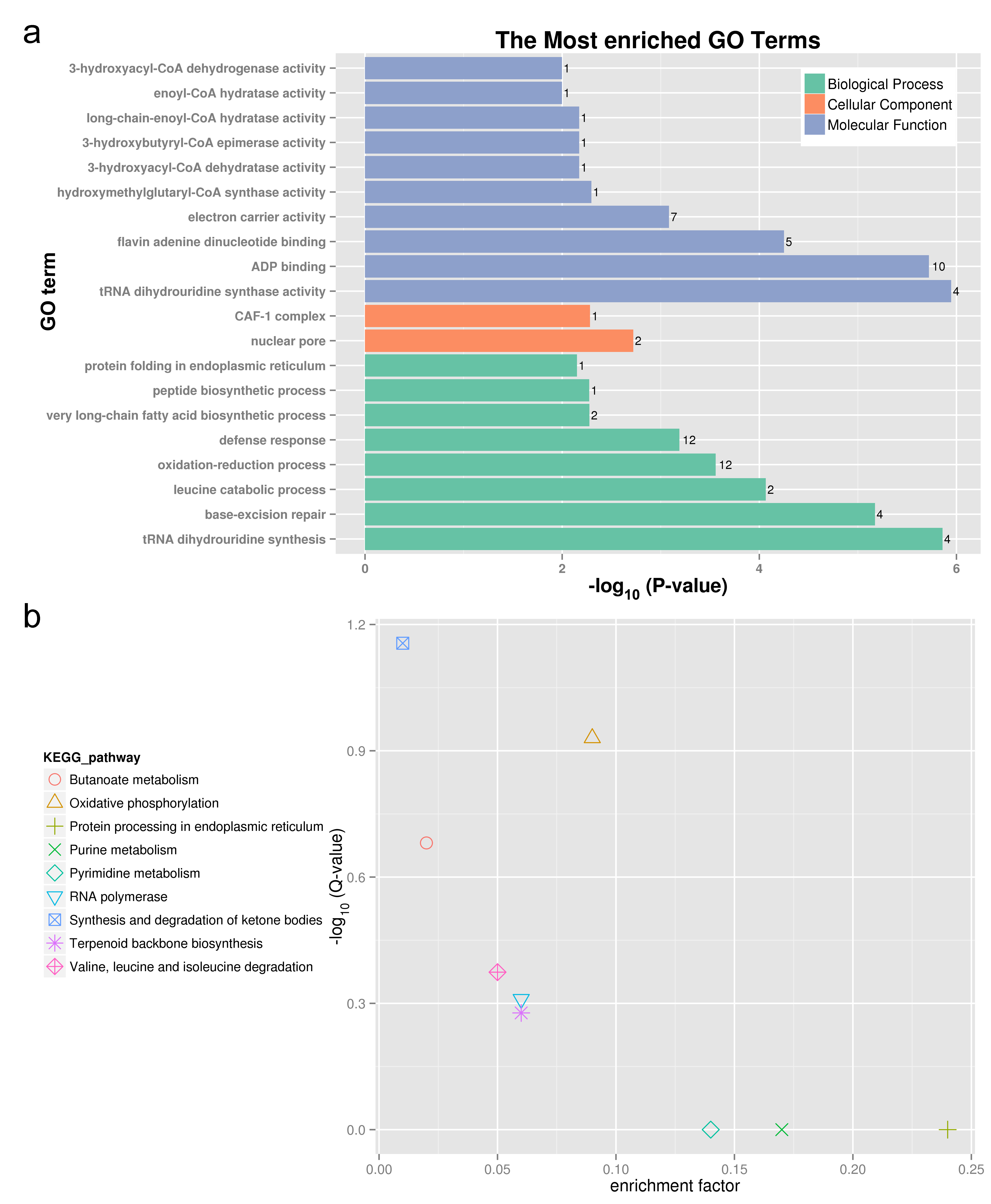

Supplement: Supplementary file 4 [file Data_Sheet_1.zip › Image 12.TIF]

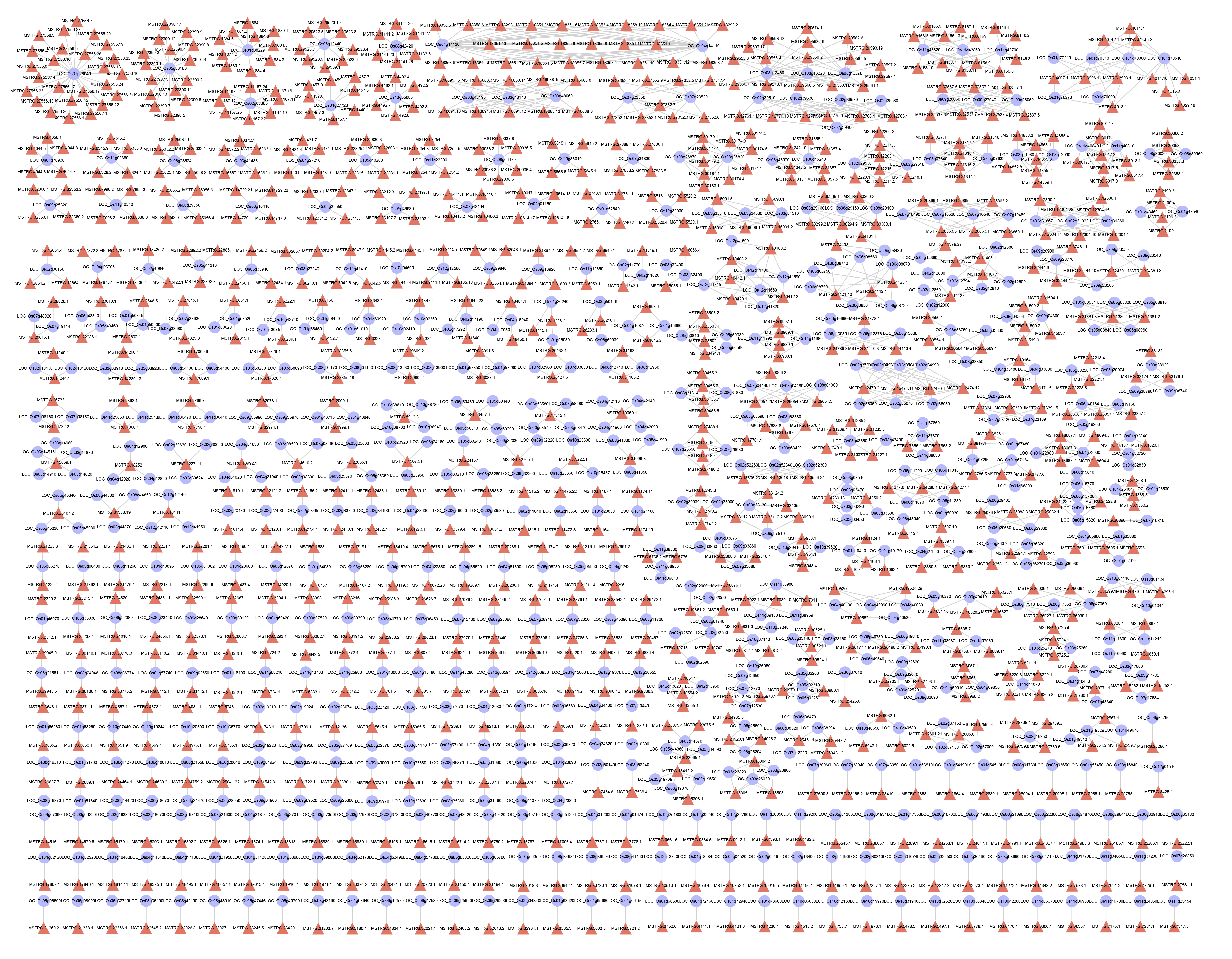

Supplement: Supplementary file 4 [file Data_Sheet_1.zip › Image 13.TIF]

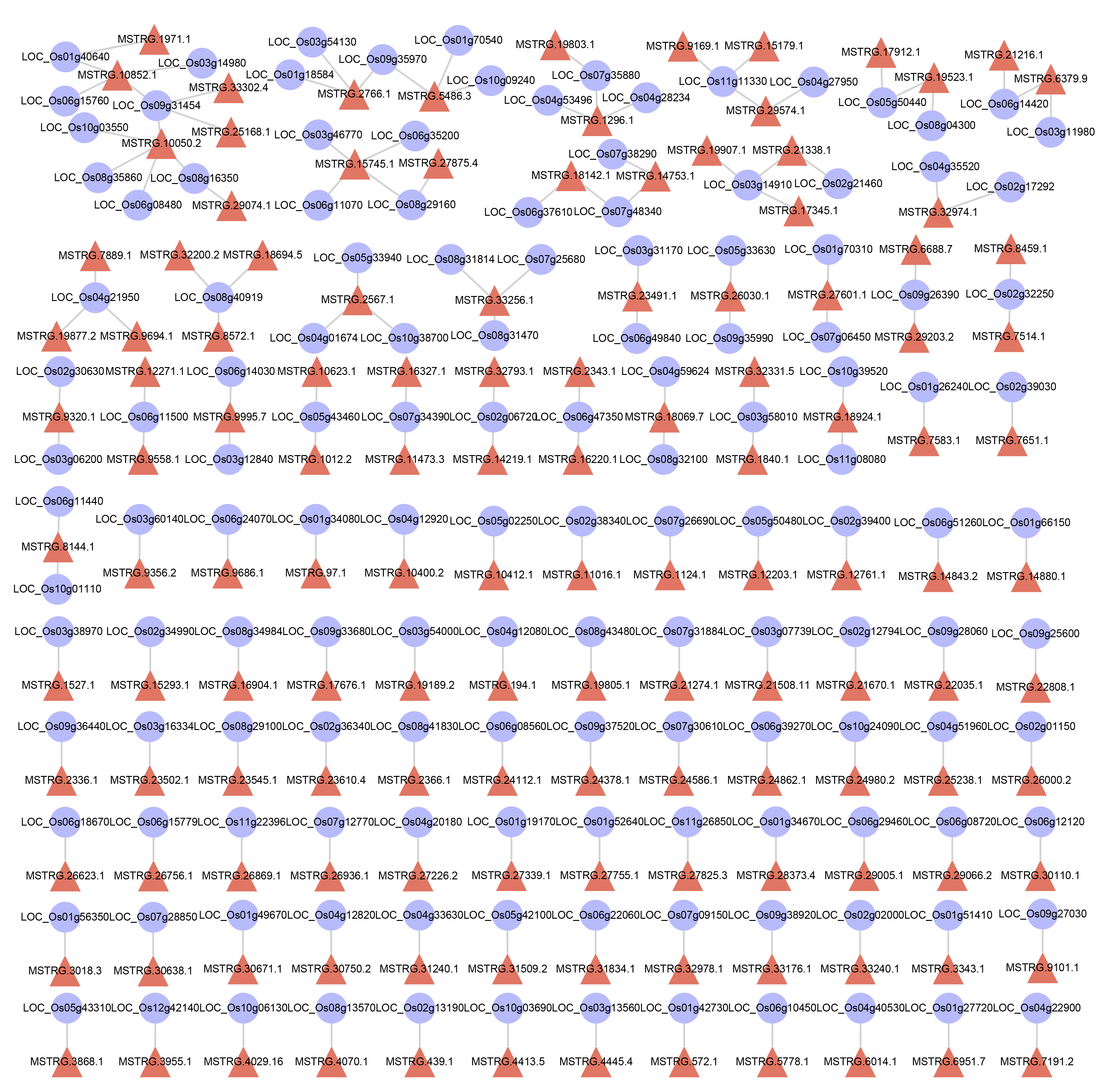

Supplement: Supplementary file 4 [file Data_Sheet_1.zip › Image 14.TIF]

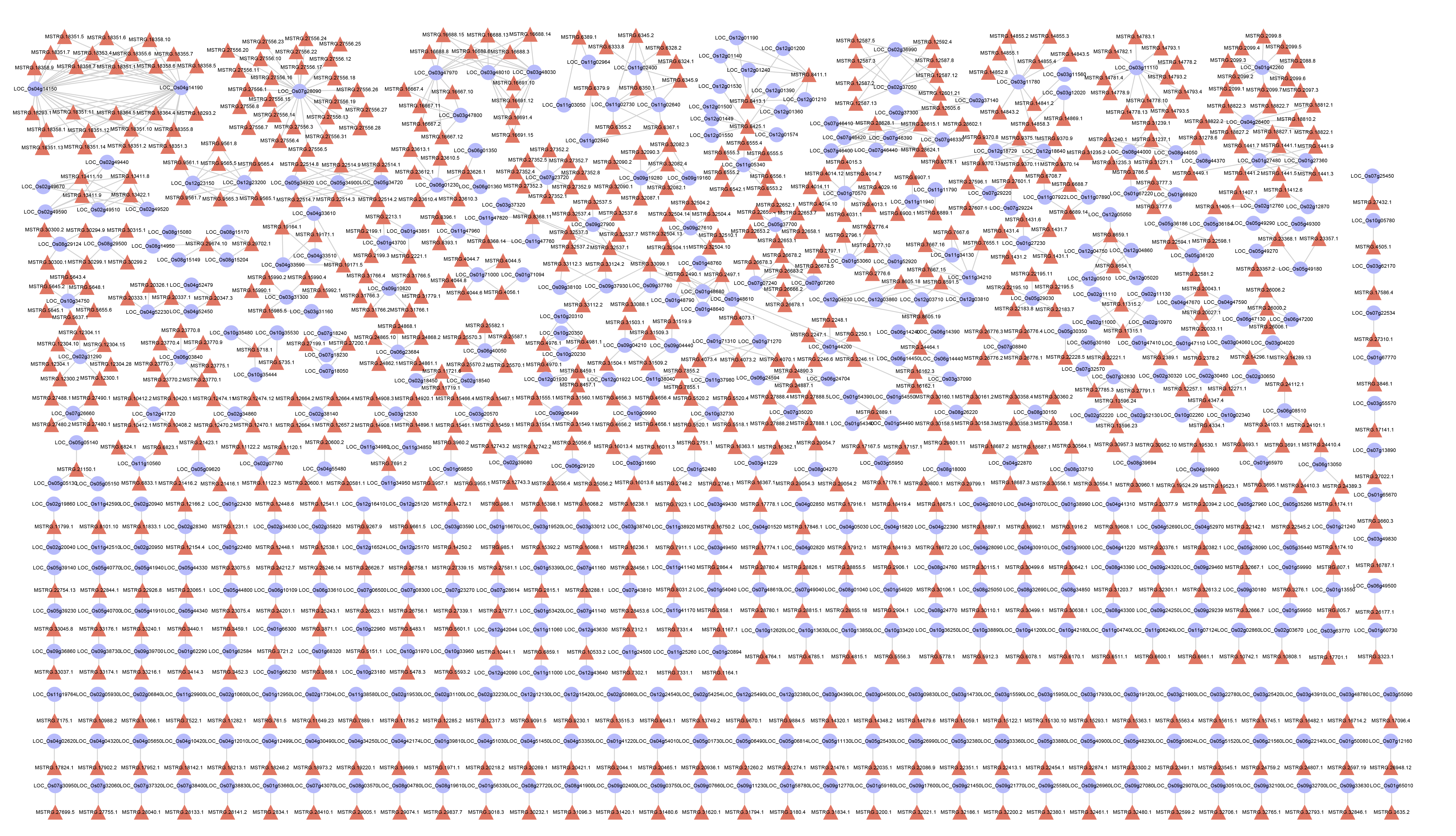

Supplement: Supplementary file 4 [file Data_Sheet_1.zip › Image 15.TIF]

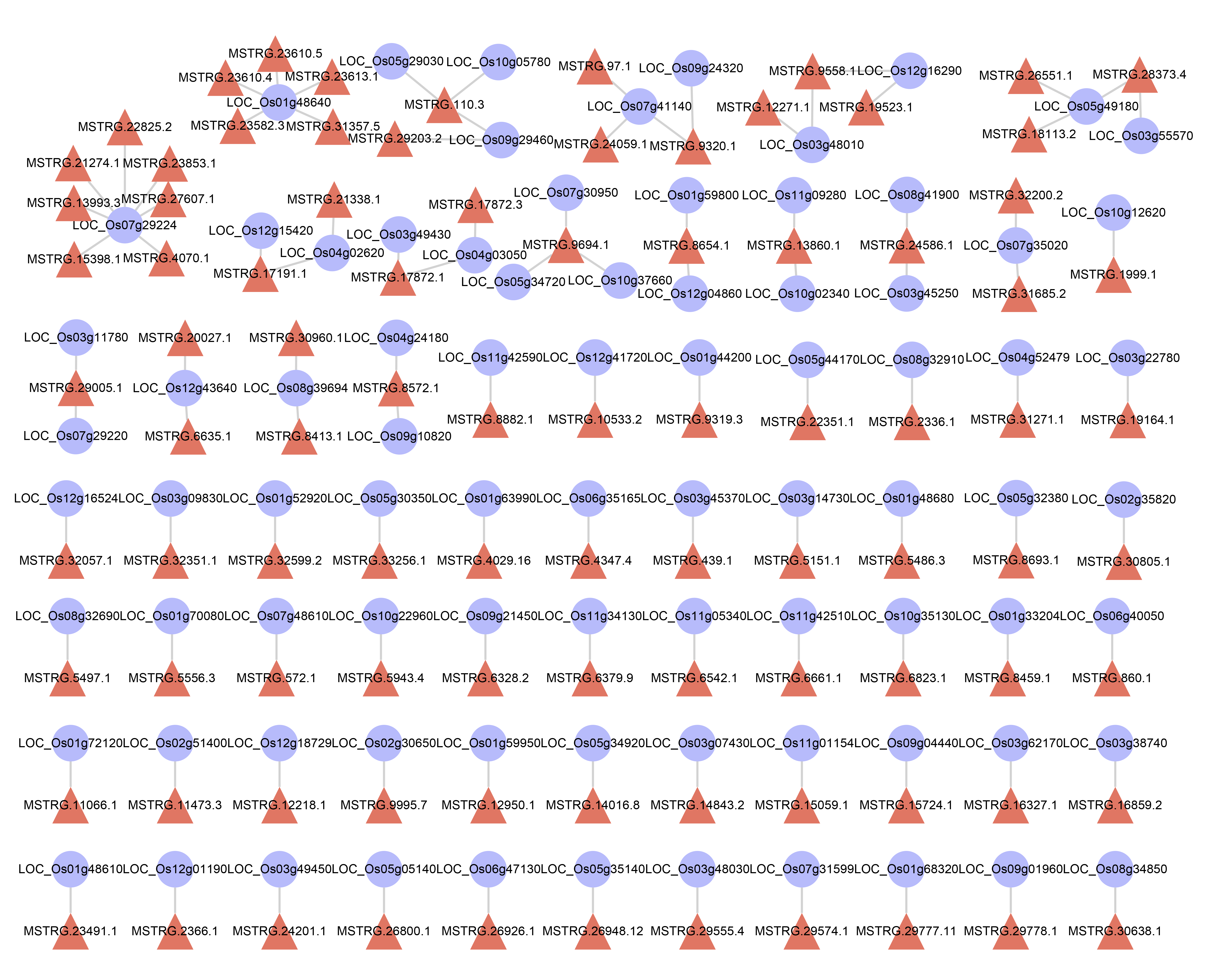

Supplement: Supplementary file 4 [file Data_Sheet_1.zip › Image 16.tif]

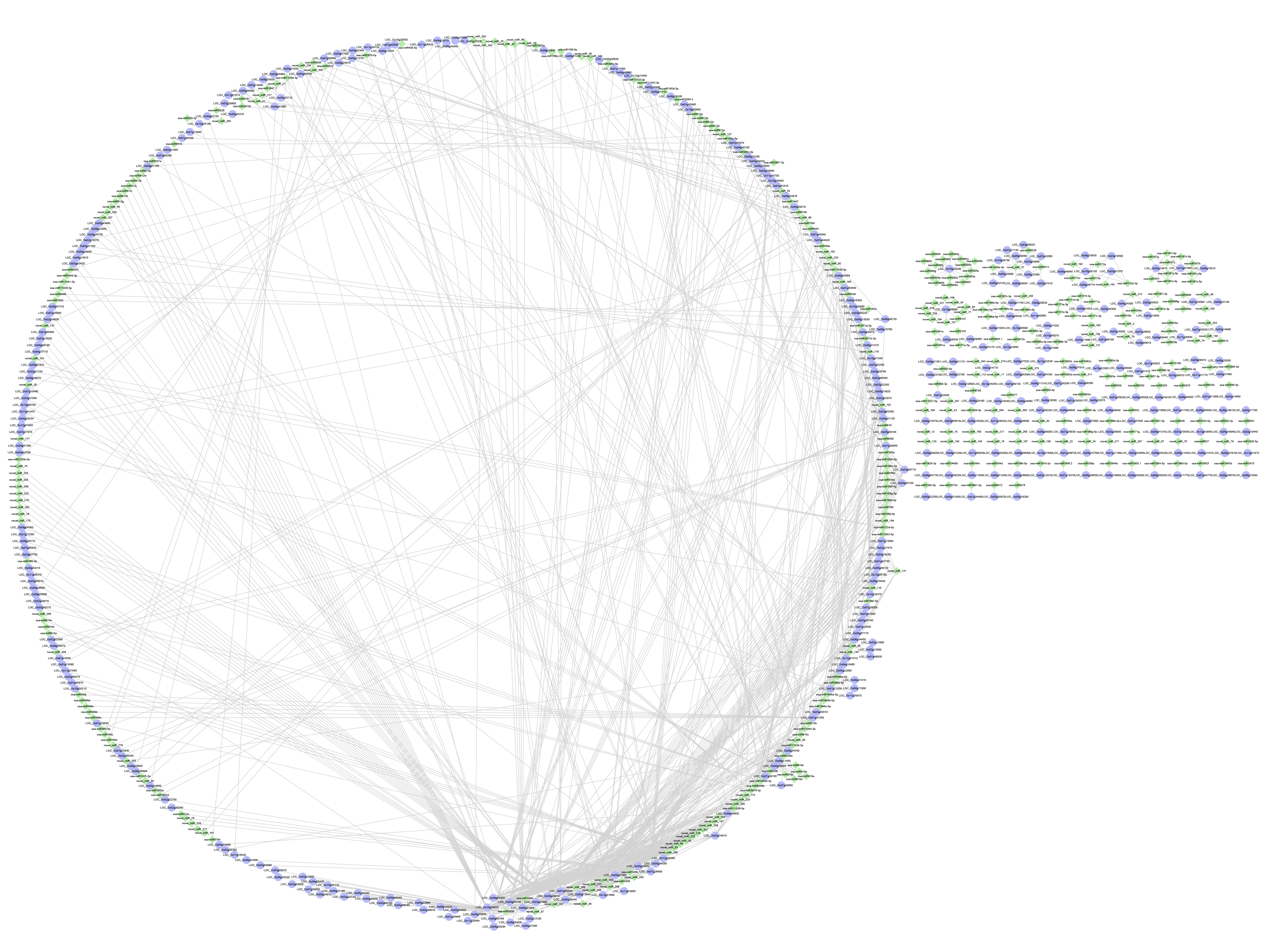

Supplement: Supplementary file 4 [file Data_Sheet_1.zip › Image 17.tif]

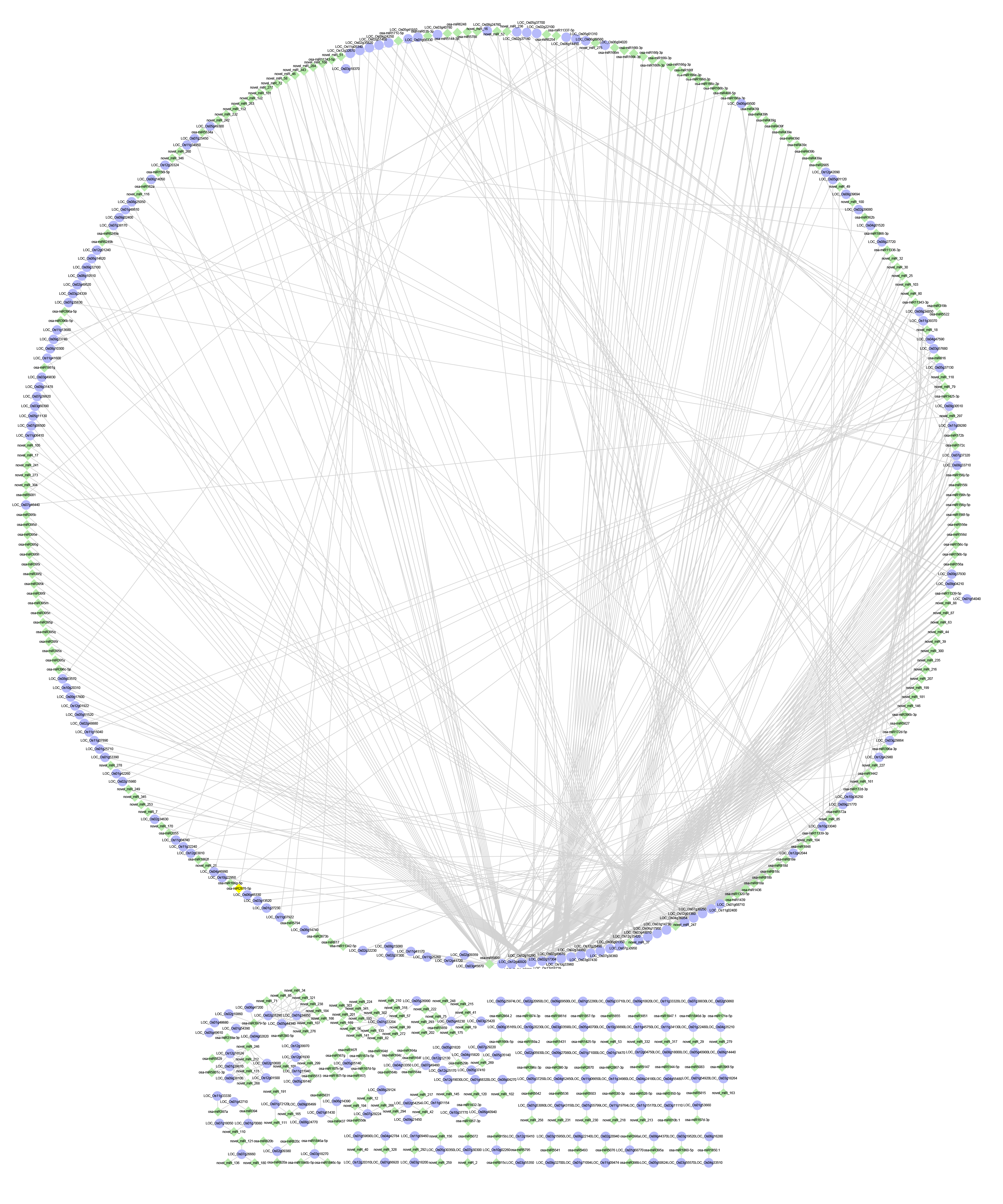

Supplement: Supplementary file 4 [file Data_Sheet_1.zip › Image 18.tif]

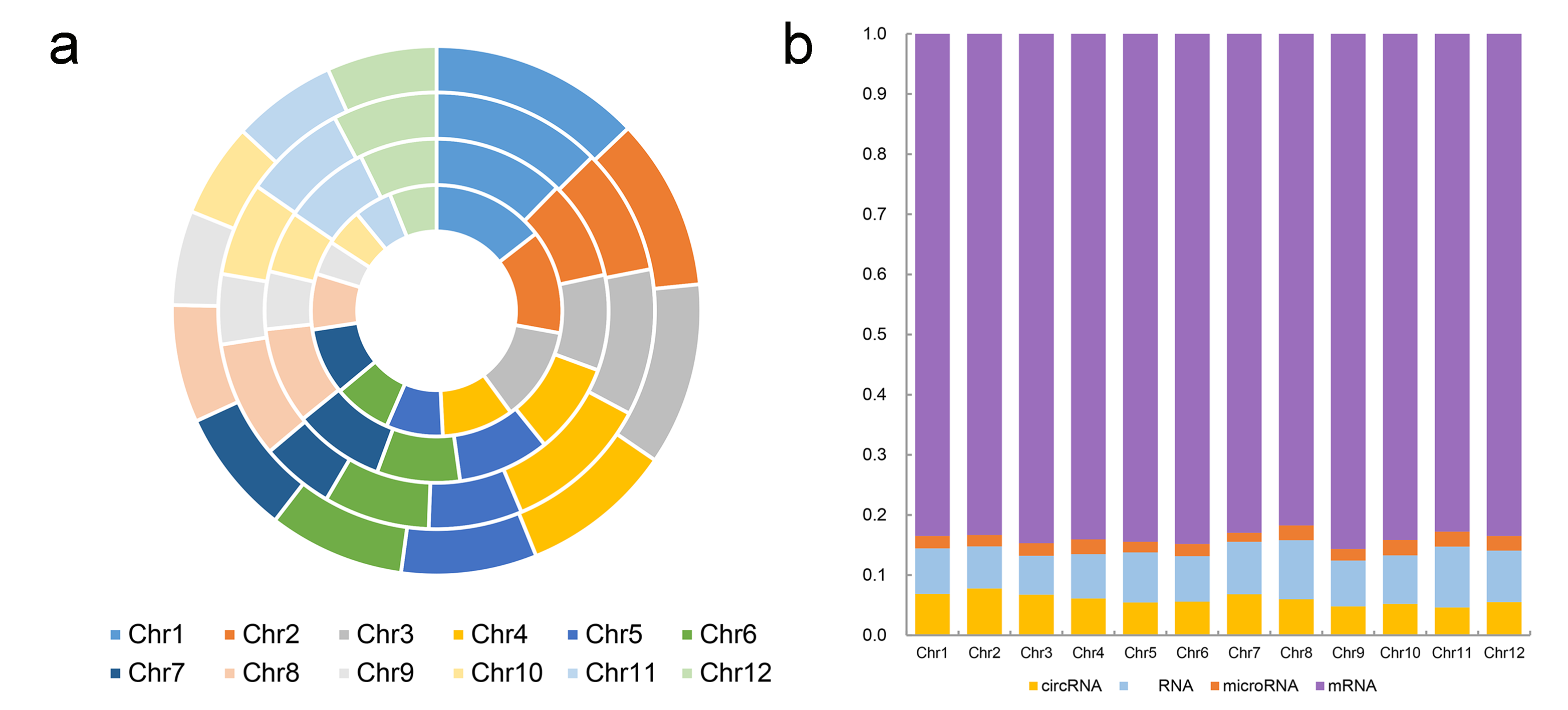

Supplement: Supplementary file 4 [file Data_Sheet_1.zip › Image 2.TIF]

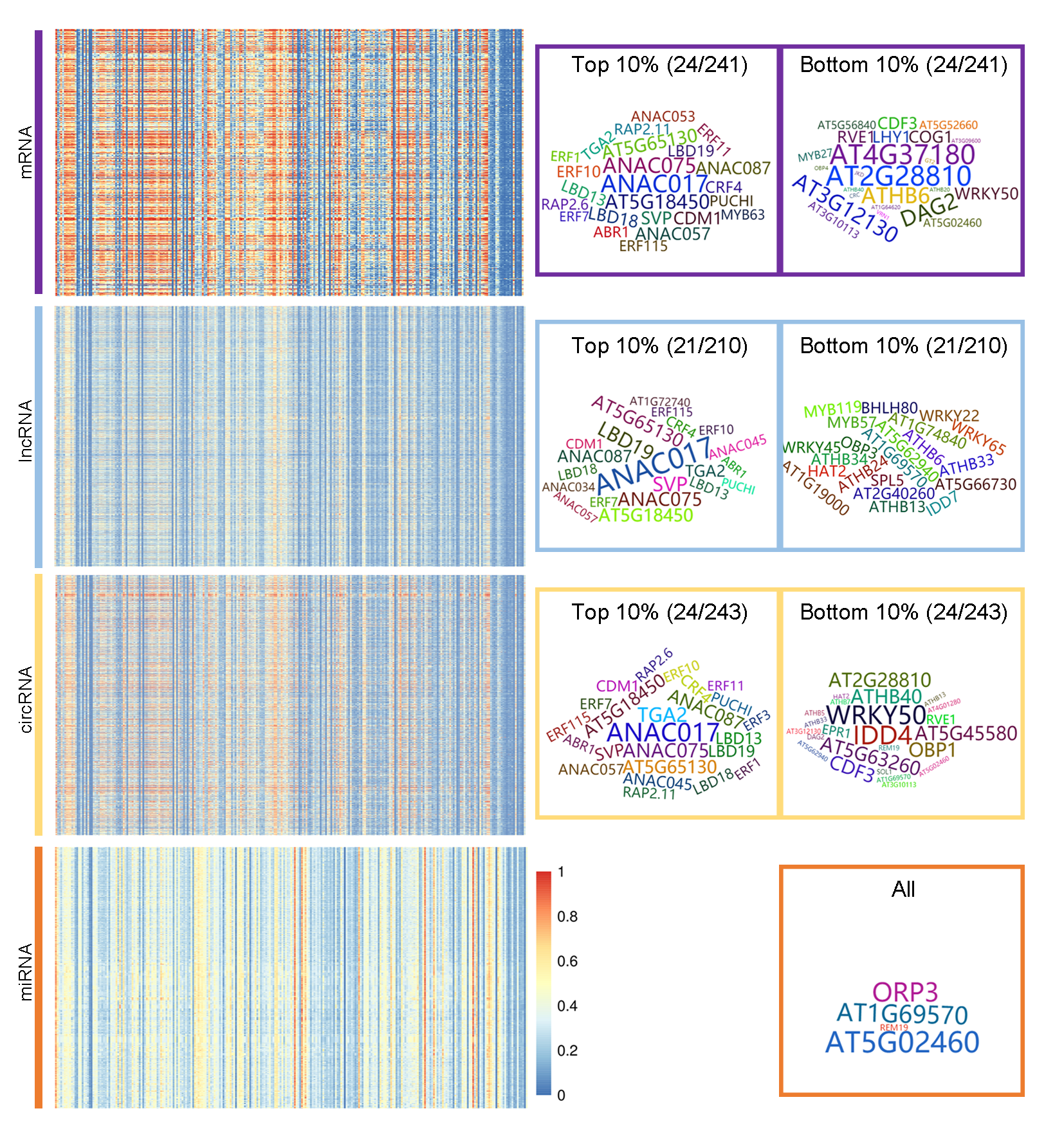

Supplement: Supplementary file 4 [file Data_Sheet_1.zip › Image 3.TIF]

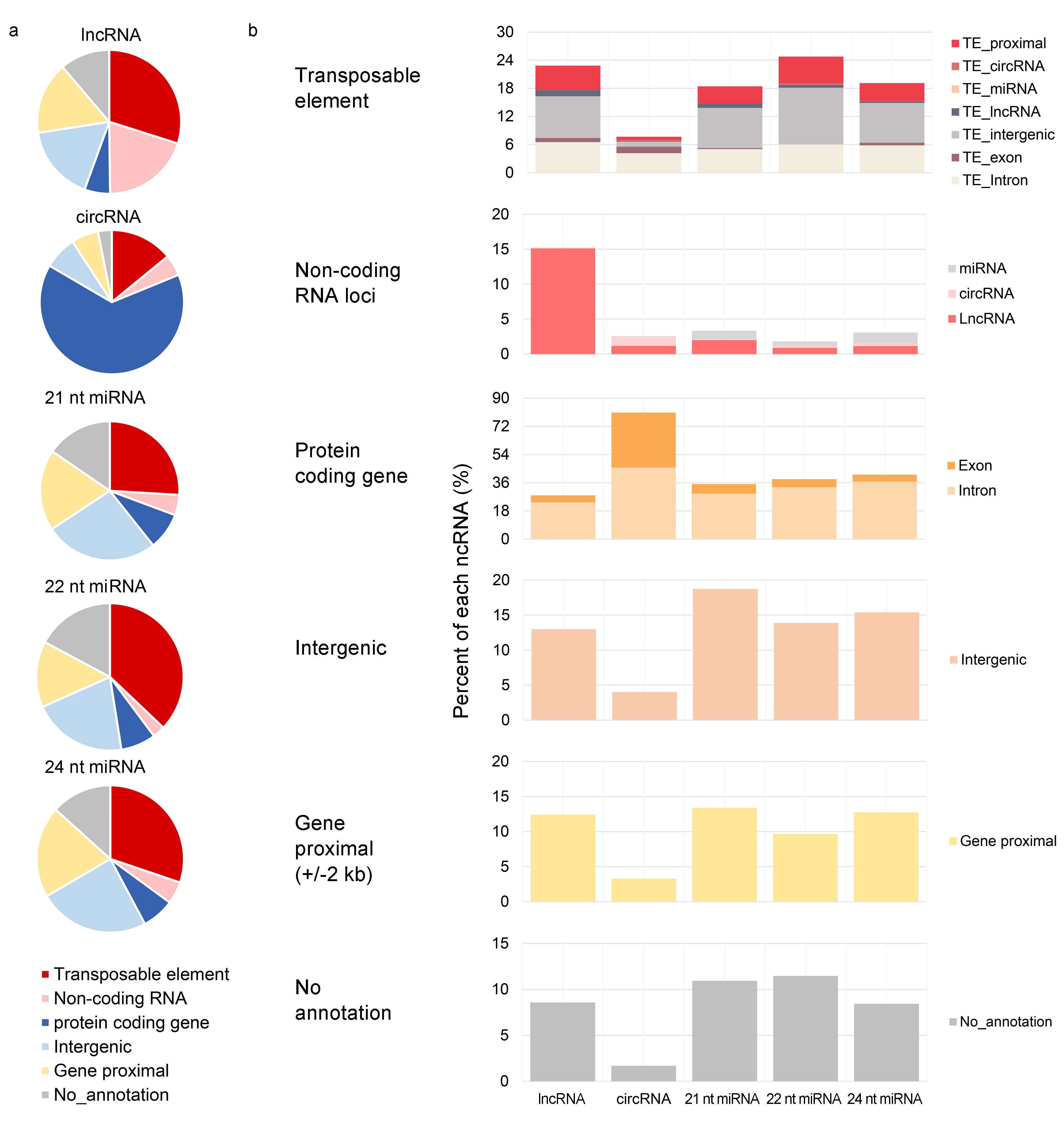

Supplement: Supplementary file 4 [file Data_Sheet_1.zip › Image 4.TIF]

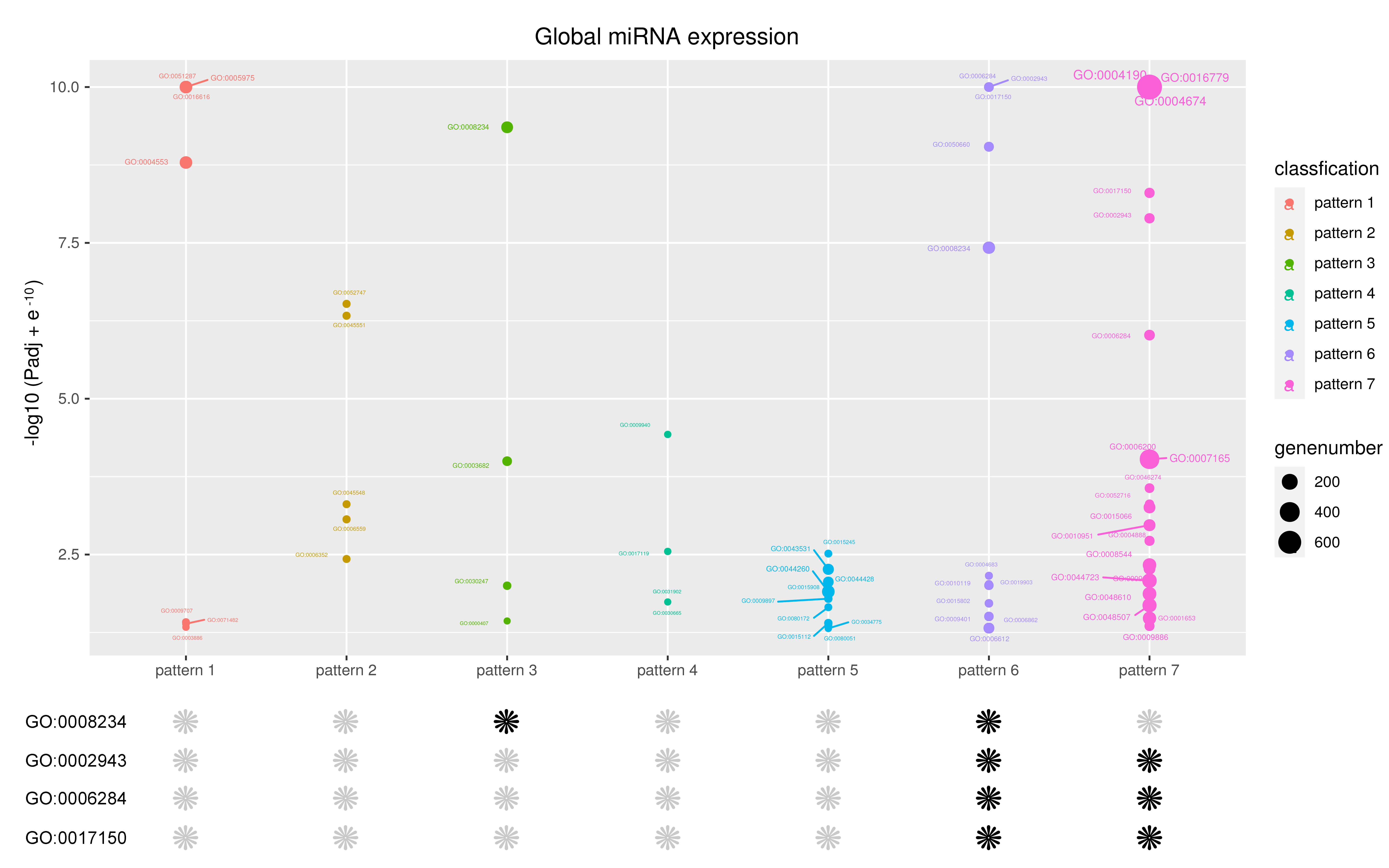

Supplement: Supplementary file 4 [file Data_Sheet_1.zip › Image 5.TIF]

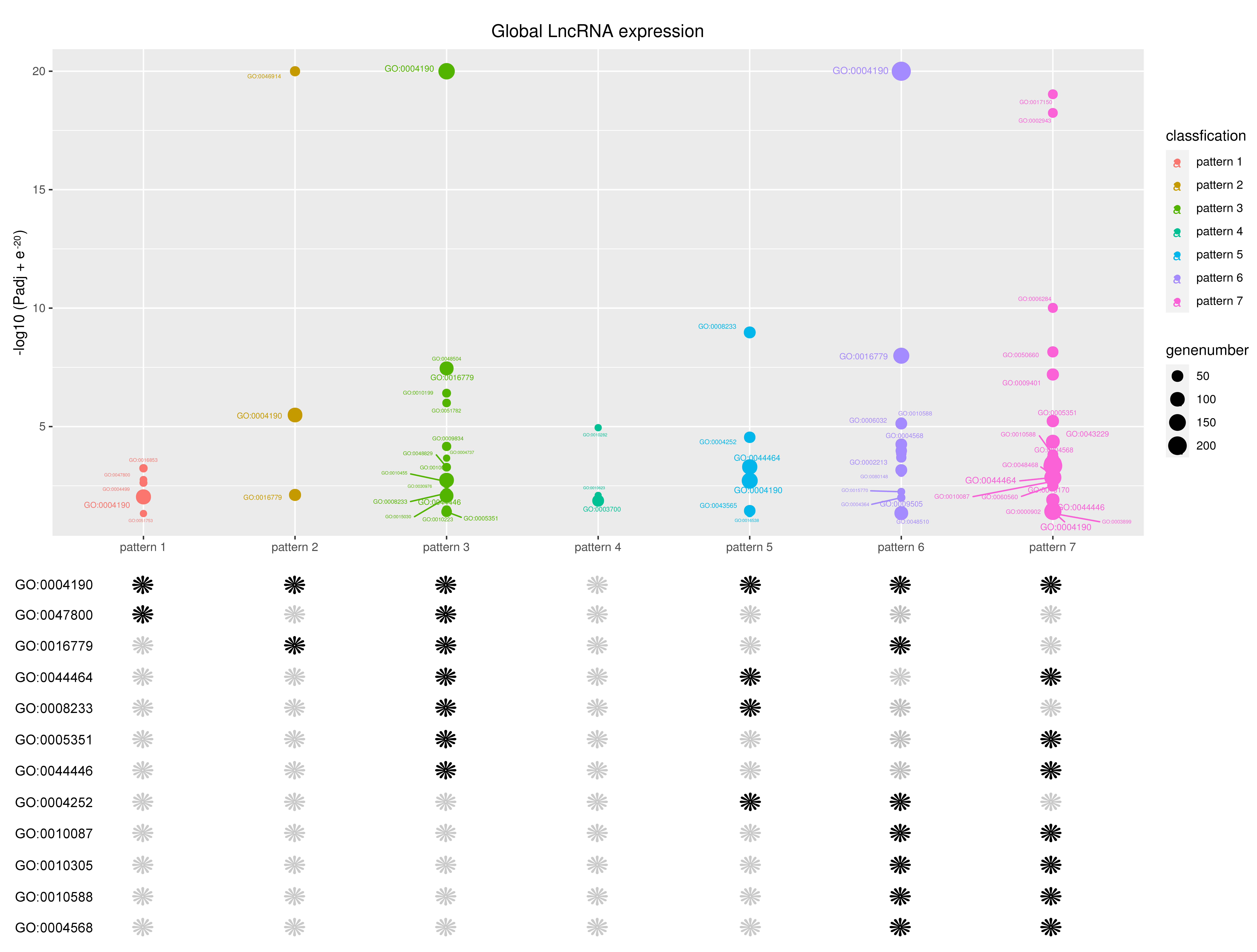

Supplement: Supplementary file 4 [file Data_Sheet_1.zip › Image 6.TIF]

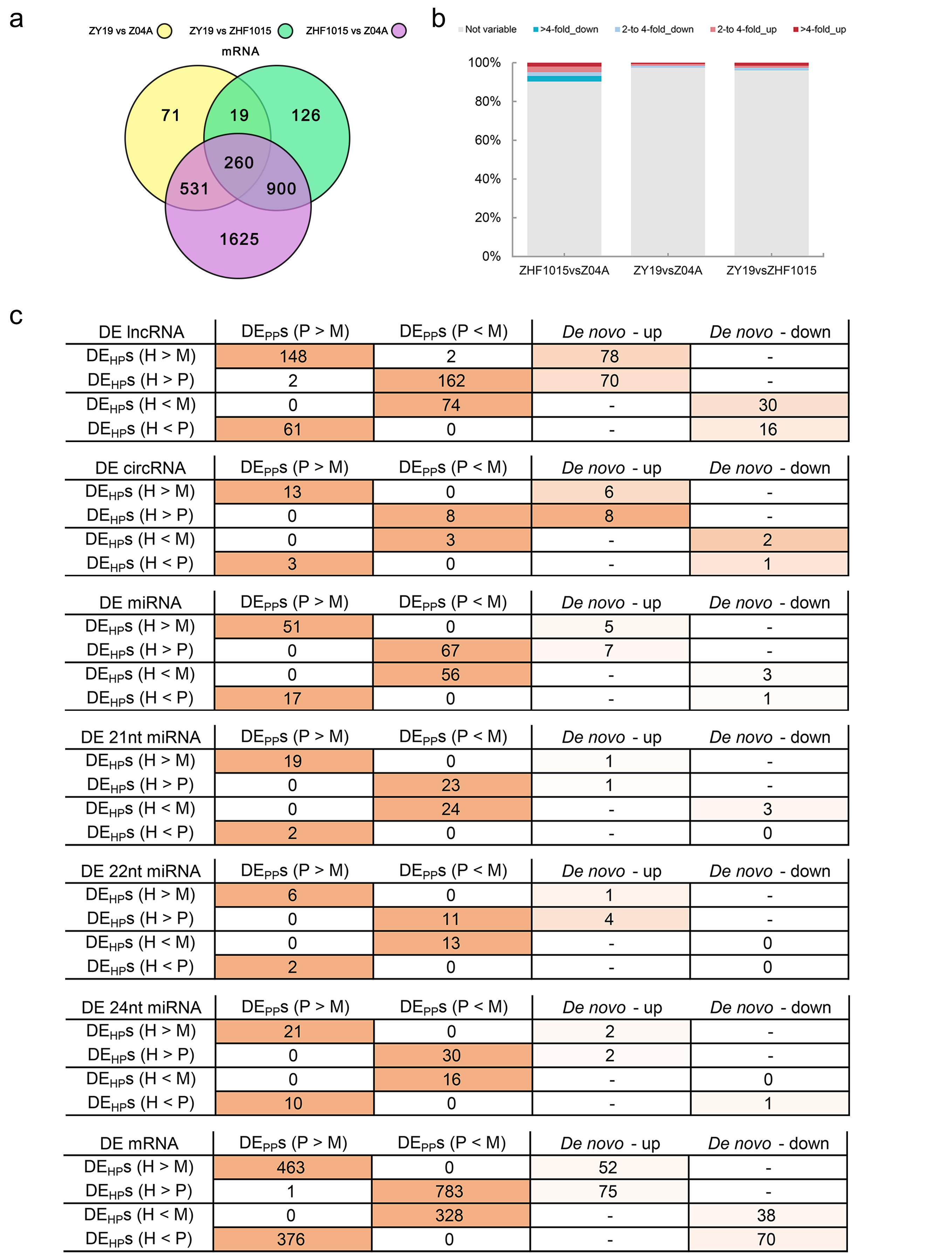

Supplement: Supplementary file 4 [file Data_Sheet_1.zip › Image 8.TIF]

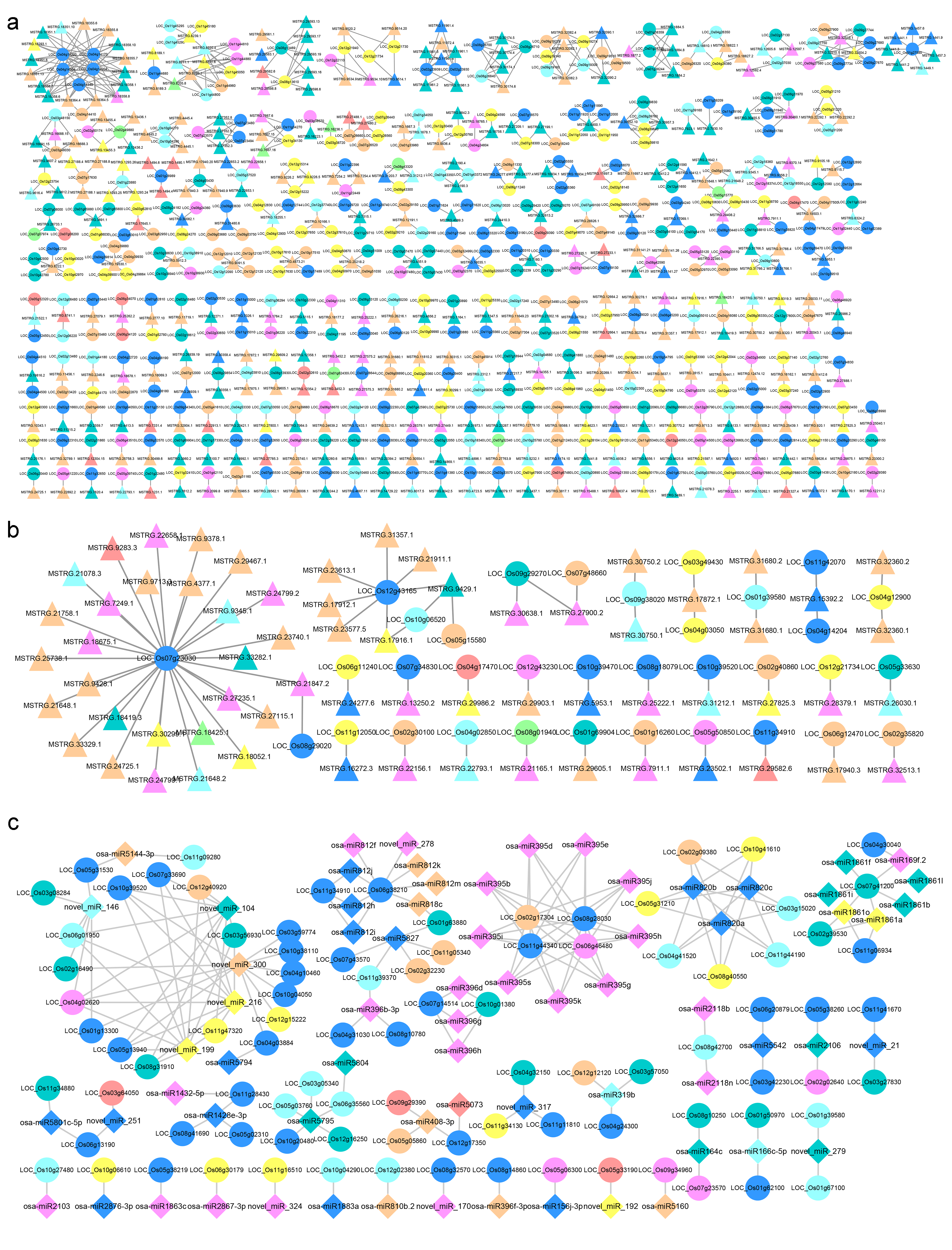

Supplement: Supplementary file 4 [file Data_Sheet_1.zip › Image 9.TIF]
